# Ionic regulation of thylakoid membrane architecture: Mg^2+^-driven destacking and restacking visualized

**DOI:** 10.1093/plphys/kiag101

**Published:** 2026-02-27

**Authors:** Jarne Berentsen, Erwin Hogeveen, Emilie Wientjes

**Affiliations:** Laboratory of Biophysics, Wageningen University & Research, Wageningen 6708 WE, The Netherlands; Laboratory of Biophysics, Wageningen University & Research, Wageningen 6708 WE, The Netherlands; Laboratory of Biophysics, Wageningen University & Research, Wageningen 6708 WE, The Netherlands

## Abstract

The thylakoid membrane houses the complexes involved in the light-harvesting reactions of photosynthesis. In plants, this membrane is intricately folded into cylindrical grana stacks, connected by stroma lamellae. This architecture allows for the lateral segregation of photosystem II in the grana and photosystem I in the stroma lamellae. The thylakoid ultrastructure is dynamic and can change in response to light and other environmental cues, allowing for regulation of the light-harvesting reactions. Isolated thylakoid membranes in vitro can reversibly destack and restack depending on the concentration of cations such as Mg^2+^. However, it is currently unknown how this destacking and restacking is possible, given the complex thylakoid architecture. Here, we combine fluorescence spectroscopy with expansion and electron microscopy to investigate the reversible Mg^2+^-dependent stacking of *Arabidopsis thaliana* thylakoids in vitro. Our data suggest that the Mg^2+^ concentration determines the segregation of photosystem I and photosystem II in the thylakoid membrane, regardless of prior status (stacked or destacked). Furthermore, the microscopy results show that thylakoids under fully destacked conditions still retain loose grana-like structures. The loose nature of this thylakoid architecture likely allows the intermixing of the photosystems. Furthermore, our data suggest thylakoids undergo structural reorganizations upon Mg^2+^-induced restacking. While complete thylakoid destacking and restacking do not occur in vivo, our results offer insights into how subtle changes in ionic conditions could influence energy distribution and protein mobility through local modulation of membrane stacking.

## Teaser

Thylakoids destack and photosystems intermix upon Mg^2+^ removal; Mg^2+^ re-addition leads to structural reorganisations, as observed via expansion microscopy.

## Introduction

Photosynthesis in plant cells occurs in the chloroplasts, which contain the stromal fluid and the thylakoid membrane. The thylakoid is a crowded membrane, where proteins account for roughly 70% of the total area (Kirchhoff 2014). These proteins include the pigment-protein light-harvesting complexes (LHCs) and photosystems (PSs) that absorb sunlight and drive photosynthesis. The absorbed light energy is used for charge separation in the reaction centers (RCs) of PSI and PSII. Consequently, these photosystems work together to convert the light energy into chemical energy in the form of the energy carriers NADPH and ATP. These energy carriers are used in the Calvin–Basham–Benson cycle to fix carbon dioxide (Blankenship 2021).

The thylakoid is a continuous membrane, folded into cylindrical granum stacks and stroma lamellae that connect these stacks. The current model suggests that the stroma lamellae wrap helically around the grana (Paolillo Jr 1970; Bussi et al 2019). This folding allows for the accommodation of a large membrane surface in a small volume (Nevo et al 2009). The 2 thylakoid domains differ both in structure and contents: PSII is mainly located in the grana (Staehelin and van der Staay 1996; Albertsson 2001), while PSI is confined to the stroma lamellae and grana end membranes (Wietrzynski et al 2025), as its bulky stromal protrusions restrict access into the appressed grana membranes (Nevo et al 2012). Though this structure is well established, it is currently unclear why the thylakoid is folded into such an intricate structure (Trissl and Wilhelm 1993; Nevo et al 2012; Pribil et al 2014).

Nevertheless, several reasons have been proposed explaining why this structure offers advantages over a homogeneous thylakoid membrane. For instance, the spatial separation between the PSs reduces the likelihood of direct excitation energy transfer from PSII to PSI, a process referred to as spillover (Kim et al 2005; Pribil et al 2014). The grana also provide a structure to concentrate PSII, sharing their LHCs and optimizing light harvesting (Trissl and Wilhelm 1993; Chow et al 2005). Furthermore, the spatial separation of the photosystems allows for dynamic regulation of the light-dependent reactions via architectural changes and protein organization rearrangements. For example, the LHC that is usually connected to PSII (LHCII) can migrate toward PSI under conditions where PSI absorption is less optimal, or back when PSII absorption is less optimal. These state transitions are regulated by the phosphorylation of LHCII and result in a reestablished excitation balance between the photosystems (Lemeille and Rochaix 2010; Tikkanen et al 2011). In addition, the spatial separation could protect PSII from premature degradation (Chow et al 2005; Herbstová et al 2012; Puthiyaveetil et al 2014).

The thylakoid membrane is negatively charged, due to negatively charged lipids (Kansy et al 2014), acidic protein groups (Nakatani et al 1978), and posttranslational modifications such as phosphorylation (Puthiyaveetil et al 2017). As such, 2 main mechanisms of thylakoid stacking have been identified: (i) electrostatic attraction between the negatively charged thylakoid membrane and the positively charged N-terminus of LHCII, which stabilizes thylakoid stacks (Carter and Staehelin 1980; Standfuss et al 2005; Albanese et al 2020), and (ii) charge screening by cations such as Mg^2+^, which reduces electrostatic repulsion and promotes membrane adhesion via van der Waals forces (Barber 1980b; Kim et al 2005; Puthiyaveetil et al 2017) or salt bridge formation between adjacent membranes (Wan et al 2014). It is well-established that cations are needed to stabilize the thylakoid structure, as electron microscopy (EM) (Izawa and Good 1966; Jennings et al 1978; Burke et al 1979; Briantais et al 1984; Bassi et al 1985; Kirchhoff et al 2007; Kiss et al 2008) and confocal scanning light microscopy (CSLM) (Rumak et al 2010) studies have shown cation-dependent thylakoid destacking and restacking in isolated thylakoid membranes. Especially Mg^2+^ seems to influence the stacking to a large degree (Jennings et al 1978; Rumak et al 2010).

Mg^2+^-dependent thylakoid destacking and restacking can be followed by pulse-amplitude modulation (PAM) fluorometry (Kirchhoff et al 2007; Kiss et al 2008) and 77 K fluorescence emission (Burke et al 1979; Krause et al 1983; Kirchhoff et al 2007; Kiss et al 2008). In PAM experiments, the destacking of thylakoids by depletion of Mg^2+^ is indicated by a decrease in the fluorescence signal of PSII in the presence of 3-(3,4-dichlorophenyl)-1,1-dimethylurea (DCMU) and low actinic light. Under these conditions, the RCs of PSII are closed, leading to the maximum fluorescence of PSII. The decrease in signal upon destacking is caused by the disruption of the PSII-LHCII complex and intermixing of PSI and PSII upon destacking (Kiss et al 2008). This stage is characterized by an increase in the 77 K fluorescence emission of PSI compared to PSII, signifying spillover between the PSs as a result of the intermixing (Krause and Weis 1991; Kirchhoff et al 2007; Van der Weij-de Wit et al 2007; Pribil et al 2014). This intermixing of PSI and PSII upon destacking has been observed using freeze-fracture EM (Kirchhoff et al 2007) and is further supported by the equal distribution of PSI and PSII between thylakoid membrane fractions (Anderson and Vernon 1967; Van der Weij-de Wit et al 2007; Trotta et al 2025). When Mg^2+^ is reintroduced, the room temperature PSII fluorescence increases while the relative 77 K PSI fluorescence decreases, signifying that the lateral segregation of the PSs is restored. This segregation and subsequent thylakoid restacking is mediated by van der Waals–operated attraction (Puthiyaveetil et al 2017) and the ionic screening of the negative charges, causing PSII to aggregate (Rojdestvenski et al 2002). These ordered assemblies promote attractive interactions between opposing membranes, facilitating the formation of stacked grana structures (Standfuss et al 2005; Kirchhoff et al 2007; Albanese et al 2020). This shows that changes to the thylakoid architecture can be prompted by the ionic environment. Since the thylakoid membrane is a highly dynamic membrane, this ionic environment-dependent self-organization of the thylakoid architecture could be beneficial for the regulation of the light-harvesting reactions (Kirchhoff et al 2007).

It remains unclear how the intricate 3D architecture of the thylakoid can undergo cation-dependent destacking and restacking. This ability of the thylakoid membrane is puzzling, as the grana and stroma lamellae membranes mix during destacking, leading to the loss of the lateral segregation of PSI and PSII, which is reversed during restacking (Briantais et al 1984; Kirchhoff et al 2007). Thus far, the thylakoid structure during destacking and restacking has been mainly studied with thin-section EM, which images a fraction of the thylakoid in 2D. Some 3D information on this process has been obtained with confocal microscopy (Rumak et al 2010), but the resolution is too low to fully observe the thylakoid membranes. Recently, we have employed expansion microscopy (ExM) to look at the 3D structure of the thylakoid membrane of spinach and *Arabidopsis thaliana* (*A. thaliana*) with ∼60 nm spatial resolution, allowing us to separate grana from stroma lamellae (Bos et al 2024; Berentsen et al 2025). Here, we combine ExM with PAM fluorometry and 77 K fluorescence emission measurements to follow the thylakoid de- and restacking in vitro. We validate the structures observed using ExM with transmission EM (TEM). We show that the thylakoid fluorescence characteristics are dependent on the Mg^2+^ concentration, regardless of the previous (stacked or destacked) status of the thylakoid. Furthermore, thylakoids retain some recognizable structure under destacking conditions. Finally, we suggest that thylakoids undergo a structural reorganization upon restacking of the membranes, where new grana are formed. These observations provide insight into the possible role of Mg^2+^ in thylakoid maintenance and dynamics.

## Results

In this work, we followed thylakoid destacking and restacking using various techniques. First, PAM fluorescence was used to follow the room temperature PSII fluorescence during destacking and restacking. In these experiments, DCMU was present to close all PSII RCs under weak actinic light (55 µmol m^−2^ s^−1^). As a result, we observed the maximum PSII fluorescence during the measurements. PSI and PSII intermix upon thylakoid destacking, allowing for excitation energy transfer from PSII to PSI, which reduces the room temperature fluorescence of PSII (Barber 1980b). The PSII fluorescence intensity therefore reflects the stacking state of the thylakoid. However, the addition of actinic light and DCMU also results in a decrease of the fluorescence signal when no destacking occurs (Fig. S1). This decrease is most likely due to photoinhibition caused by the combination of DCMU and actinic light (Aro et al 1993; Rantala et al 2020). Correction for this photoinhibition is not possible, as we are not certain the degree of photoinhibition in the destacked or (partially) restacked state is the same as in the stacked state.

**Figure 1:**
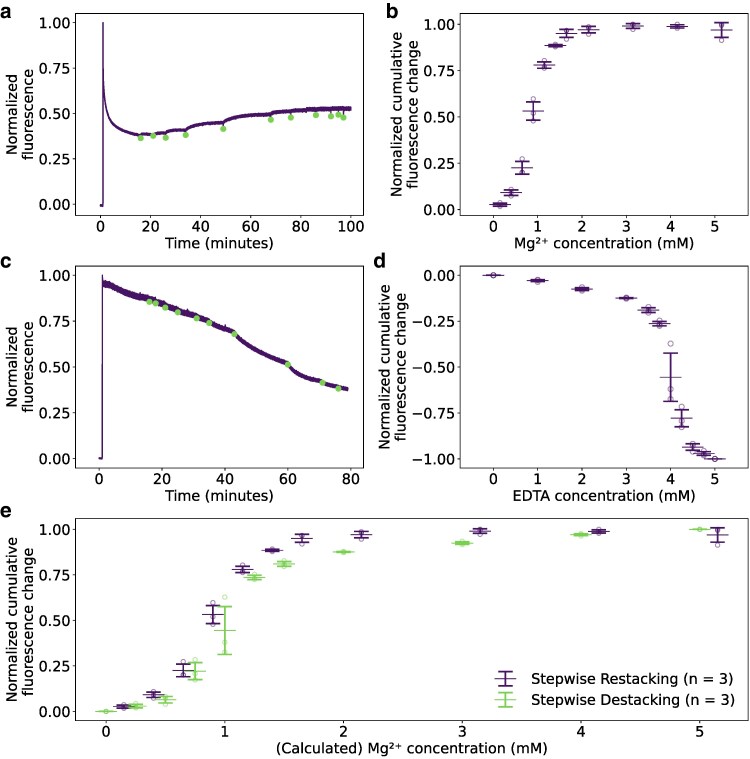
Room temperature thylakoid fluorescence upon stepwise restacking and destacking. **a)** Thylakoid fluorescence upon destacking and stepwise restacking. Thylakoids suspended in GB were diluted in a buffer containing EDTA. After 15 min under these destacking additions, titration steps of MgCl_2_ were carried out. The green markers indicate when MgCl_2_ was added. **b)** Cumulative fluorescence changes after each titration step of MgCl_2_. The open circles represent individual measurements; the horizontal line with error bars represents mean ± standard deviation of 3 independent experimental runs. **c)** Thylakoid fluorescence upon stepwise destacking. Thylakoids suspended in GB were destacked with titration steps of EDTA. The green markers indicate when EDTA was added. **d)** Cumulative fluorescence changes after each titration step of EDTA. The open circles represent individual measurements; the horizontal line with error bars represents mean ± standard deviation of 3 independent experimental runs. **e)** Comparison of the cumulative fluorescence change upon stepwise restacking (purple symbols) and stepwise restacking (green symbols). The Mg^2+^ concentration was calculated assuming EDTA captures Mg^2+^ in a 1:1 molar ratio. Open circles represent independent sample preparations. The fluorescence signals in a) and c) are corrected for the fluorescence increase upon sampling. An example of this correction is provided in the Supplementary material (Fig. S9). The cumulative fluorescence changes in b) and d) were normalized to the maximum cumulative fluorescence change per experimental run prior to the determination of the mean and standard deviation.

In the case of stepwise thylakoid restacking (Fig. 1a), the thylakoids were first destacked in a buffer without Mg^2+^, but with ethylenediaminetetraacetic acid (EDTA) to capture the Mg^2+^ present in the sample. While the Mg^2+^ was removed from the sample, the fluorescence signal decreased. We tested several durations of this destacking, ranging from 5 to 90 min. We found no clear indication that a longer duration led to more destacked thylakoids (Figs. S2 and S3). To ensure thylakoids were fully destacked before stepwise restacking was started, thylakoids were subjected to the destacking conditions for 15 min for all experiments. After this destacking, Mg^2+^ was reintroduced with stepwise additions. New additions occurred when the fluorescence signal was stable. The initial additions did not increase the fluorescence to a large extent and led to a new stable fluorescence signal in a short amount of time. However, later additions led to a substantial increase in the fluorescence signal, taking multiple minutes to reach a steady-state level. The final additions led to similar changes as the initial additions, until no further increase was observed (Fig. 1a). The fluorescence signal recovered only to roughly 50% of the initial fluorescence. This can be explained by photoinhibition, as the fluorescence signal of stacked thylakoids under the same measurement conditions also substantially decreases in the same amount of time (Fig. S1).

The cumulative fluorescence changes after each addition (Fig. 1b) follow a sigmoidal-shaped curve. This curve shows that the largest changes in fluorescence signal occur around 1 mM Mg^2+^. This curve also shows that the fluorescence change plateaus after roughly 2 mM Mg^2+^, signifying that this concentration is enough to fully restack thylakoids.

Similar to the stepwise thylakoid restacking, the stepwise destacking was also followed (Fig. 1c). For this, thylakoids were diluted in a buffer containing 5 mM Mg^2+^. Initially, the signal decreased, indicating the presence of photoinhibition of PSII throughout the experiment. After 15 min, stepwise additions of EDTA were carried out to capture the Mg^2+^ and lower its concentration in the solution. Similar to the Mg^2+^ additions during the stepwise restacking, EDTA was only added when the fluorescence signal had stabilized. The fluorescence decreases after the EDTA additions mimic the fluorescence increases after the Mg^2+^ additions during the restacking: Initial additions had little impact on the fluorescence signal, but subsequent additions caused large decreases, after which further additions resulted in minimal change. This is again reflected in the sigmoidal-shaped cumulative fluorescence change (Fig. 1d).

At the pH of the experiments (pH 7.0 to 7.6), the binding constant of EDTA to Mg^2+^ (Henzl et al 2003) is much higher than the binding constant of the thylakoid membrane to Mg^2+^ (Isaakidou and Papageorgiou 1975). This indicates that EDTA efficiently captures Mg^2+^ from the thylakoid. In addition, EDTA binds Mg^2+^ in a 1:1 ratio (O’Brien et al 2015). As a result, the Mg^2+^ concentration can be estimated by subtracting the present EDTA concentration from the starting Mg^2+^ concentration. The cumulative fluorescence signal change between stepwise restacking and destacking can therefore be compared (Fig. 1e). This comparison shows that stepwise destacking and restacking follow a similar path.

**Figure 2:**
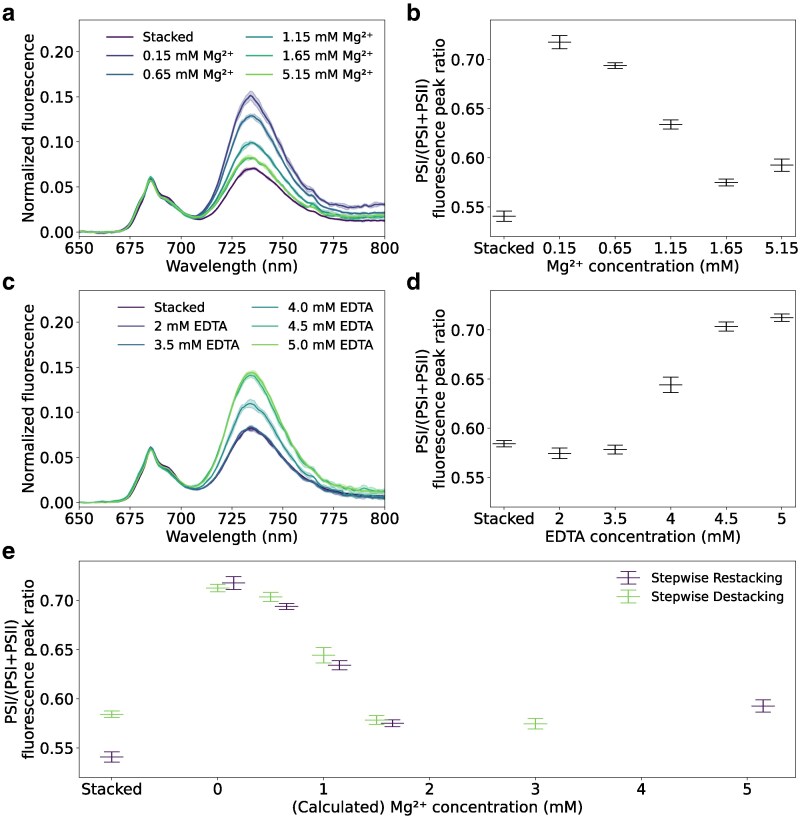
77 K fluorescence emission of thylakoids upon stepwise restacking and destacking. **a)** 77 K fluorescence emission spectra of thylakoids upon stepwise restacking with MgCl_2_. **b)** PSI over (PSI + PSII) fluorescence peak ratio of thylakoids upon stepwise restacking with MgCl_2_. **c)** 77 K fluorescence emission spectra of thylakoids upon stepwise destacking with EDTA. **d)** PSI over (PSI + PSII) fluorescence peak ratio of thylakoids upon stepwise destacking with EDTA. **e)** Comparison of the cumulative fluorescence change upon stepwise restacking (purple symbols) and stepwise restacking (green symbols). For the stepwise destacking experiment, the Mg^2+^ concentration was calculated assuming EDTA captures Mg^2+^ in a 1:1 molar ratio. The shaded area in a) and c) represents standard deviation over 5 spectra recorded from the same sample. Symbols in b), d), and e) represent means ± standard deviation from 5 recorded spectra of the same sample preparation. Spectra are representative of at least 2 independent sample preparations (Fig. S4).

The 77 K fluorescence emission spectra of thylakoids at various stages during the stepwise destacking or restacking were recorded (Fig. 2). While the fluorescence of PSI is very weak at room temperature, its fluorescence quantum yield is strongly increased at low temperature (Itoh and Sugiura 2004). The 77 K spectra show 2 major bands, namely, of PSII (around 685 nm) and of PSI (around 732 nm) (Kiss et al 2008). In the stacked thylakoids, the maximum height of these bands is comparable (Fig. 2a and c). The PSI band increased relative to the PSII band under low magnesium concentrations. This observed change is an indication of the occurrence of spillover, where the excitation energy of PSII is directly transferred to PSI (Kim et al 2005; Pribil et al 2014), thereby increasing the PSI fluorescence at the cost of PSII fluorescence. Instead, the PSI fluorescence decreased relative to PSII when Mg^2+^ was reintroduced (Fig. 2a and b). When EDTA was added to stacked thylakoids, the PSI band increased relative to the PSII band with increasing EDTA concentration (Fig. 2c and d). Similar to the cumulative fluorescence change described above, a comparison between the stepwise destacking and stepwise restacking (Fig. 2e) shows that the PSI/(PSI + PSII) 77 K emission ratio is comparable at similar Mg^2+^ concentrations, regardless of whether the sample was stepwise destacked or restacked. The 77 K fluorescence emission spectra thus show that PSI and PSII are intermixed under low Mg^2+^ concentrations. However, upon increasing the Mg^2+^ concentration, the contribution of PSI to the total fluorescence decreases, suggesting lateral segregation between the PSs. As such, it can be presumed that the Mg^2+^ concentration determines the PSII to PSI spillover and thus the lateral segregation of PSI and PSII.

**Figure 3:**
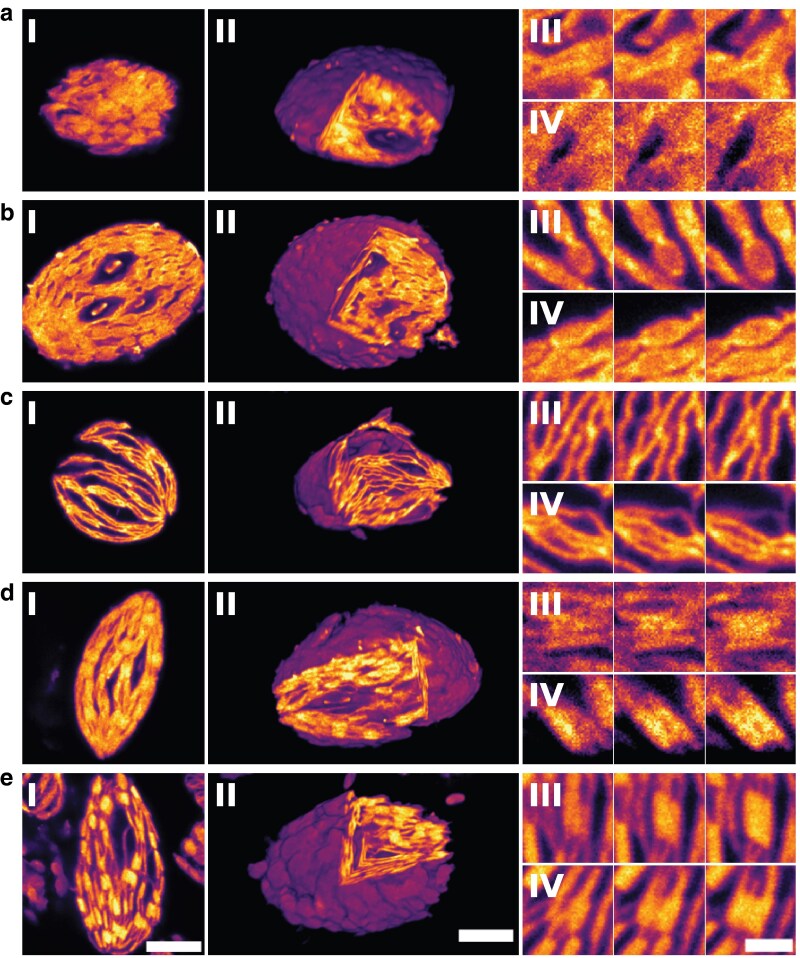
Examples of the different structural thylakoid classes found upon stepwise restacking as seen by ExM. Examples of thylakoids portraying the characteristics of the **a)** structureless, **b)** nodular, **c)** fibrous, **d)** restacked, and **e)** stacked class. Of each class, a 2D representation (I), 3D representation (II), and 2 z-montages of close-ups (III and IV) are presented. The z-montages consist of consecutive z-slices of a granum (roughly 77 nm apart, corrected for the ∼4.6× expansion). Scale bars are corrected for the expansion factor (∼4.6×). Scale bars in panels I and II represent 2 µm and is applicable to all images in those panels. The scale bar in panel IV represents 500 nm and is applicable to all images in panels III and IV. Samples were stained with an ATTO-594 NHS-ester, providing all-protein staining.

**Figure 4:**
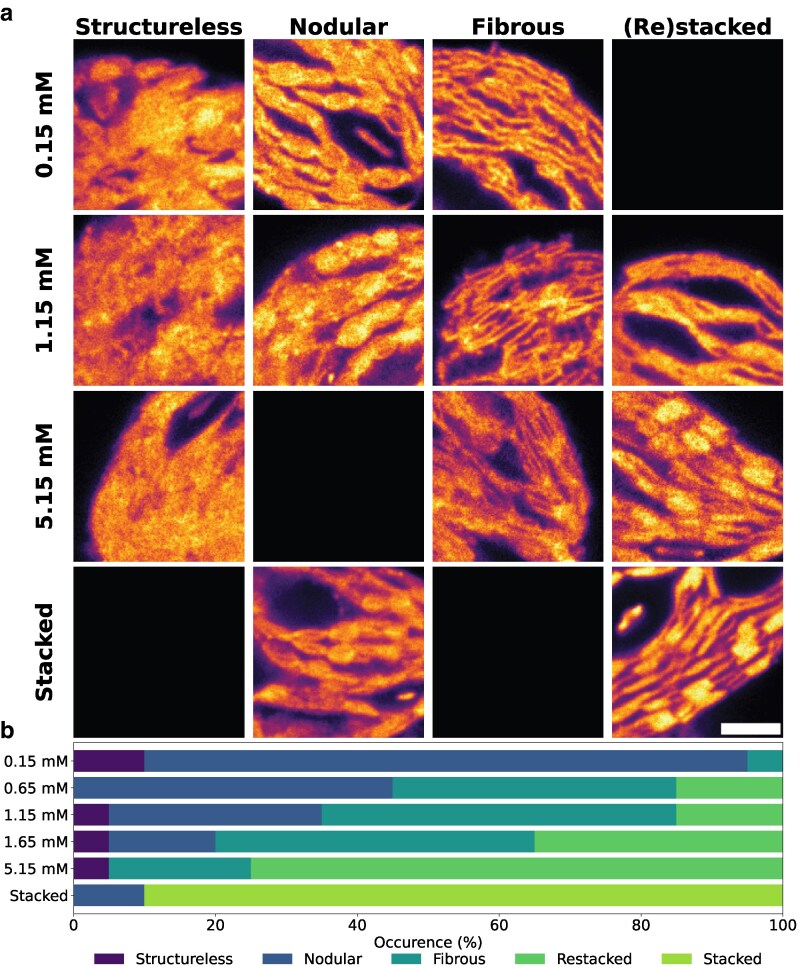
Thylakoid classes and their distribution at different Mg^2+^ concentrations. **a)** Examples of ExM images of thylakoid structure at different Mg^2+^ concentrations. Zoomed in examples of the different classes per Mg^2+^ concentration are shown. Scale bar represents 1 µm, corrected for the expansion factor (∼4.6×), and is applicable to all presented examples. Samples were stained with ATTO-594 NHS ester for all-protein visualization. The absence of an image indicates that class was not found at the Mg^2+^ concentration. Zoomed out examples are presented in Fig. S7. **b)** Distribution of the thylakoid structural classes at different Mg^2+^ concentrations as determined via visual inspection of the thylakoids. Similar trends are found across the 3 independent biological replications (Fig. S6).

Next, the various states of restacking were visualized using ExM to resolve the structure of the entire thylakoids. Based on these images, we could classify thylakoids into 5 distinct groups: structureless, nodular, fibrous, restacked, and stacked. These classes are observable in both 3D (Fig. 3) and 2D (Figs. 3 and  4a; Fig. S5). Thylakoids were classed as nodular if grana were observed, yet these grana looked ellipsoidal instead of rectangular (Fig. 3b, III and IV). Often, little to no separation was observed between the grana in thylakoids of this architecture, resulting in an architecture that resembles beads on a string. In contrast, fibrous thylakoids lacked well-defined grana stacks. Instead, they exhibited weakly stacked regions that appeared as dispersed or irregular thylakoid interspersed with stroma lamellae or possibly pairs of appressed membrane layers, termed doublets (Garty et al 2024). While some early signs of grana formation were detectable in this state, stacking was incomplete and spatially separated, resulting in a loose, filamentous architecture with a comparable overall 3D shape to other classes (Fig. 3c). Stacked thylakoids show clear grana structures. In this architecture, grana are often observed as rectangular shapes, interconnected by the stroma lamellae. We separated restacked from stacked, as restacked thylakoids show less pronounced, yet recognizable, grana compared to the stacked thylakoids. Thylakoids were classified as structureless if none of these characteristics were observed. The lack of structure is probably caused by the homogenization conditions during isolation. Those thylakoids were also imaged due to the random selection of thylakoids from a large low-resolution overview. Thylakoids in this class are therefore artifacts of the experimental procedure.

The different classes were most clearly observed in side views of thylakoids. Therefore, side views of the thylakoids during the restacking process were analyzed. Visual inspection of the 2D and 3D thylakoid images showed different distributions of the classes under different Mg^2+^ concentrations (Fig. 4b; Fig. S6). Due to the qualitative nature of the inspection, the fraction sizes are not absolute and only meant to give an indication of the structures observed at different Mg^2+^ concentrations. Nevertheless, qualitatively, a clear trend of change in distribution across the different Mg^2+^ concentrations can be observed. A large portion of the thylakoids at low Mg^2+^ concentrations displayed a nodular structure. This fraction decreased upon increasing Mg^2+^ concentration, while the fractions of thylakoids with fibrous or restacked structure increased.

**Figure 5:**
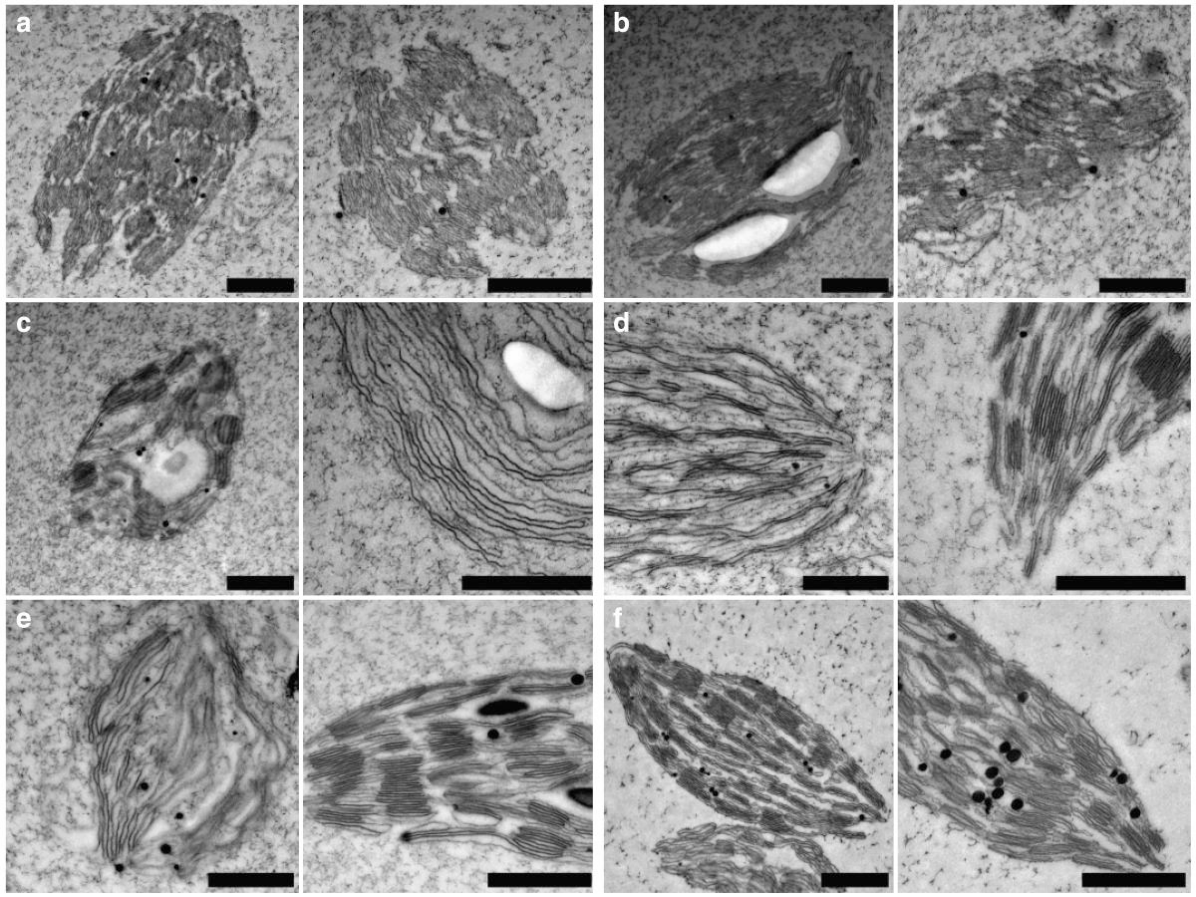
EM micrographs of thylakoids under different Mg^2+^ concentrations. Images are representative of destacked thylakoids in the presence of **a)** 0.15 mM, **b)** 0.65 mM, **c)** 1.15 mM, **d)** 1.65, or **e)** 5.15 mM Mg^2+^. **f)** Stacked thylakoids as isolated. The nodular thylakoid structure is seen in a), b), and the left image of c). The fibrous structure is observed in the right image of c) and the left images of d) and e). The restacked class is visible in the right images of d) and e). Presented EM images were made with 8,000× to 15,000× magnification. Scale bars represent 1 µm. Samples were stained with 2% uranyl acetate.

To validate the structures observed using ExM, thylakoids in the same states of restacking as visualized with ExM were imaged using TEM (Fig. 5). In these micrographs, individual thylakoid membranes are visible, as the higher resolution of TEM compared to ExM allows for the observation of finer structural details. The general characteristics found using ExM are also observed in the EM micrographs. At low Mg^2+^ concentrations, the nodular architecture is apparent (Fig. 5a, b, and the left image of c). At intermediate Mg^2+^ concentrations, thylakoids appear to have a more fibrous architecture (Fig. 5c, right, and Fig. 5d and e, left images). At high Mg^2+^ concentrations, thylakoids exhibit restacking (Fig. 5d and e, right images), resembling the structure in the stacked state (Fig. 5f).

**Figure 6:**
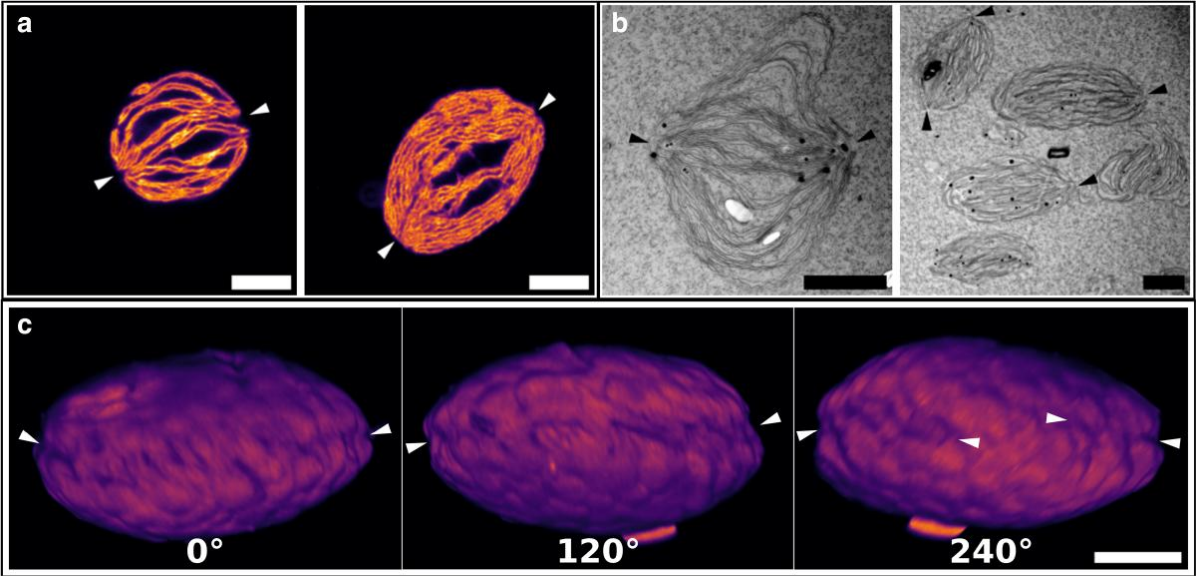
Nexus zones as observed with ExM and EM. Examples of nexus zones in **a)** ExM (stained with ATTO-594 NHS-ester; the right image also appears in Fig. S5 and in a zoomed-in view in Fig. 4) and **b)** EM images (stained with 2% uranyl acetate; a zoomed-in version of the left image is also shown in the right panel of Fig. 5c). Arrowheads indicate where the nexus zones are located. **c)** 3D representation of a thylakoid at 1.15 mM Mg^2+^, as recorded with ExM. The thylakoid was rotated in 120° intervals to observe the presence of the nexus zones around the perimeter of the thylakoid. Arrowheads indicate the crevasse-like band formed by the nexus zones create and its approximate orientation. At 240°, a region is found where the nexus zone is not clearly visible. Scale bars represent 2 µm and are corrected for the expansion factor (∼4.6×) in a) and c). For c), the scale bar is applicable to all panels.

Both ExM and EM revealed distinct regions where thylakoid membranes appear to converge or remain interconnected (indicated with arrowheads in Fig. 6). We refer to these regions as nexus zones. These zones are present in all classes, but are most easily observed in the fibrous thylakoid configuration. Some thylakoids exhibit a single nexus zone, while others display 2. The nexus zones appear to restrict membrane remodeling along the axis connecting them, while allowing dimensional changes perpendicular to this axis (Fig. 6b, left panel). This may explain why the overall ellipsoid shape of the thylakoid is preserved even under destacking conditions; although membranes can swell in height (minor axis), lateral expansion (major axis) appears constrained by these nexus zones. Importantly, the changes in shape described here reflect biological reorganization during destacking and restacking and should not be confused with the isotropic physical expansion introduced during ExM sample preparation.

To explore whether the presence of nexus zones correlates with thylakoid shape, we measured the major (width) and minor (height) axes. Although subtle differences were observed across configurations, we found no strong correlation between Mg^2+^ concentration or structural class and thylakoid aspect ratio (Fig. S7). This suggests that expansion along the minor axis is also limited, likely due to the strong interconnectedness of the thylakoid membrane network. In 3D reconstructions from ExM images (Fig. 6c), nexus zones appear as crevasse-like structures partially encircling the thylakoid. Interestingly, we also observed regions lacking such features, indicating structural heterogeneity within and between thylakoids.

## Discussion

The thylakoid membrane is an intriguing structure with a unique architecture. As with other biological membranes, there is a tight relation between the structure and function of this membrane (Kirchhoff 2013; Perez-Boerema et al 2024; Wietrzynski et al 2025). This structure–function relationship is reflected in the dynamic remodeling of thylakoid architecture in response to environmental cues, thereby optimizing the spatial organization of the photosynthetic complexes to support efficient light harvesting and electron transport (Wood et al 2018, 2019; Li et al 2020; Garty et al 2024). The organization and function of the thylakoid membrane are dynamically regulated through processes such as ion transport, protein phosphorylation, and membrane remodeling (reviewed in eg, Johnson and Wientjes (2020) and Messant et al (2021)). For example, ion levels, including the Mg^2+^ concentration, are regulated in the thylakoid membrane (Kaňa and Govindjee 2016; Kunz et al 2024). Although the effects of a change in Mg^2+^ concentration in vivo are not clear, research on isolated thylakoids has shown that Mg^2+^ depletion leads to reversible destacking of the thylakoid (Jennings et al 1978; Burke et al 1979; Bassi et al 1985; Kirchhoff et al 2007; Kiss et al 2008). These in vitro systems can give us more insight into the effect of the Mg^2+^ concentration on the thylakoid structure.

In the research presented here, we used a combination of ExM, TEM, PAM fluorometry, and 77 K fluorescence emission to follow the destacking and restacking of isolated *A. thaliana* thylakoids. While TEM, PAM fluorometry, and 77 K fluorescence are often used to study the thylakoid membrane (eg Burke et al (1979), Krause et al (1983), Kirchhoff et al (2007), and Kiss et al (2008)), ExM can provide information about the full 3D structure of the thylakoid (Bos et al 2024; Berentsen et al 2025). Although the resolution of ExM (∼60 nm (Chen et al 2015; Bos et al 2024)) is considerably lower than the resolution of TEM (∼0.5 nm (Kisielowski et al 2008)), this 3D aspect, combined with shorter sample preparation and reduced need for specialist equipment and expertise, results in a high-throughput method to visualize thylakoid membranes.

Both the PAM fluorometry and 77 K fluorescence emission show similar characteristics at similar Mg^2+^ concentrations, regardless of whether the sample was destacking or restacking. Upon depleting the Mg^2+^ concentration, the PAM signal decreased. The signal was recovered by additions of Mg^2+^ (Fig. 1). At low Mg^2+^ concentrations, the 77 K fluorescence emission showed an increase in the signal from PSI that is lessened when Mg^2+^ is reintroduced (Fig. 2). Previous research has shown that this of PSI fluorescence increase happens concurrently with the intermixing of the photosystems after Mg^2+^ removal (Hodges et al 1987; Kirchhoff et al 2007; Trotta et al 2025). This indicates that the short distance between the photosystems leads to energy spillover between the photosystems. Upon re-addition of Mg^2+^, the relative intensity of the PSI signal decreases again. Furthermore, it has been observed that restacked thylakoids display similar 77 K fluorescence emission and lateral segregation of the photosystems as stacked thylakoids (Kirchhoff et al 2007). This suggests that the Mg^2+^ concentration determines the degree of lateral segregation of the photosystems. In addition, a part of the change in 77 K fluorescence can be caused by the movement of LHCII between the photosystems, as has been observed during state transitions, as this also changes the relative PSI vs PSII 77 K fluorescence (Allen and Forsberg 2001; Tikkanen et al 2006).

It has been proposed that thylakoid destacking under low-salt conditions occurs in 3 steps (Staehelin 1986). The destacking starts by (i) separation of the appressed membranes in the grana, followed by (ii) the increase of the diameter of the connection between grana and stroma lamellae, leading to intermixing of PSI and PSII, and finally (iii) the complete unfolding of grana. Kirchhoff et al (2007) found evidence supporting this model using fluorescence measurements and freeze-fracture EM of destacked thylakoids. While our ExM and EM data (Figs. 3 to 5) confirm that the grana are destacked under Mg^2+^-deficient conditions, we still observe substantial structural retention. Rather than fully unfolding, the destacked grana often remain discernible as discrete bead-like domains. This architecture likely arises from reduced electrostatic screening, leading to repulsion between the stacked membranes (Barber 1982). Nevertheless, residual structural elements, such as the connection between grana and stroma lamellae, appear to preserve the overall grana layout. In addition, we observe that the stroma lamellae largely disappear under these conditions, with their membrane fractions becoming partly integrated into the grana. This redistribution facilitates the intermixing of the PSs and thus spillover, while still retaining a recognizable, albeit modified, thylakoid architecture.

**Figure 7:**
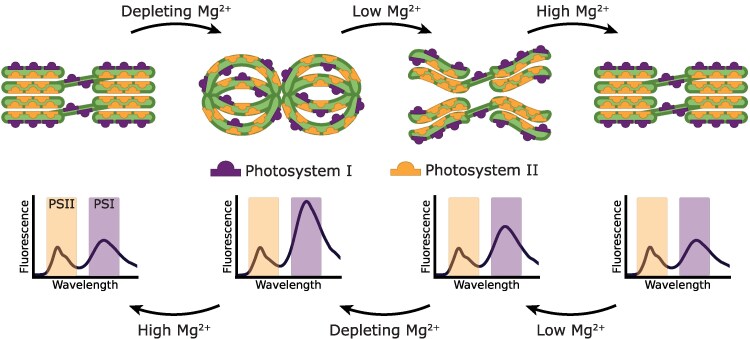
Model of the Mg^2+^-dependent thylakoid destacking and restacking. PSI and PSII are laterally segregated in the native state, with PSII inside the grana and PSI in the stroma lamellae. In this stacked state, the grana end membranes contain only PSI (Wietrzynski et al 2025). Upon depletion of Mg^2+^, the grana assume a nodular shape and the PSs intermix, leading to spillover between the PSs. When Mg^2+^ is reintroduced, PSII starts to aggregate, excluding PSI and reducing spillover. Further increasing the Mg^2+^ concentration results in the full lateral segregation of the PSs and the reformation of grana. Incremental decreasing the Mg^2+^ concentration by means of EDTA chelation increases the spillover between the PSs.

Upon increasing the Mg^2+^ concentration, more thylakoids assumed a fibrous or restacked architecture. In the initial stages, the fraction of fibrous thylakoids increased while the fraction of nodular thylakoids decreased. The fraction of restacked thylakoid increased at a later stage, concomitant with the decrease in the fibrous thylakoid fraction. This suggests that thylakoids, upon increasing Mg^2+^ concentrations, undergo a transition from the nodular state to the fibrous state, to eventually reach the restacked state. Due to the qualitative nature of our classification, the distribution only gives an indication of the relative presence of the different classes in each sample. However, this overall trend remains evident. Our 77 K fluorescence emission spectra (Fig. 2) show a decrease in the fluorescence of PSI compared to PSII upon increasing the Mg^2+^ concentration, thereby approaching the stacked state. This suggests that increasing the Mg^2+^ concentration increases the lateral segregation of PSI and PSII (Kirchhoff et al 2007). However, grana formation—as observed in the restacked thylakoids—can only occur when enough Mg^2+^ is present to screen the negative charges of the thylakoid membrane (Fristedt et al 2010; Rumak et al 2010). This suggests that the Mg^2+^-induced restacking transitions through 3 phases, as illustrated in Fig. 7. Prior to restacking, (i) thylakoids are in a destacked state with a nodular architecture, where PSI and PSII are intermixed. This architecture leads to spillover between the PSs (Barber 1980a, 1980b, 1982; Kim et al 2005; Kirchhoff et al 2007; Pribil et al 2014). Upon increasing the Mg^2+^ concentration, (ii) PSI and PSII undergo some lateral segregation, leading to the formation of PSII-enriched patches. Computational studies have indeed suggested that PSII can aggregate under intermediate ionic strengths (Rojdestvenski et al 2002). In this state, spillover is reduced due to the partial segregation of the PSs. Further increasing the Mg^2+^ concentration leads to (iii) the screening of the negative charges of the thylakoid, allowing for the formation of grana structures and full lateral segregation of the PSs.

The EM micrographs show thylakoids at different Mg^2+^ concentrations with similar structures observed in the ExM micrographs. This suggests that the ExM protocol does not alter the thylakoid structure. Both ExM and EM images reveal 1 or 2 distinct nexus zones in thylakoids (Fig. 6a and b). These nexus zones are present across classes, suggesting they are integral to the thylakoid architecture. The 3D reconstruction shows that the nexus zones often encircle the thylakoid like a band, possibly stabilizing its structure (Fig. 6c). Interestingly, these 3D reconstructions also show a local absence of the nexus zones. This local absence might explain why some side-view cross-sections only show 1 nexus zone. This suggests the thylakoid membrane could be structurally analogous to a bivalve mollusk, consisting of 2 membrane regions linked by a central junction. While we observed occasional outward deformations perpendicular to the nexus axis in the destacked state (Fig. 6b, left), we found no significant difference in the thylakoid width (major axis) or height (minor axis) for different Mg^2+^ concentrations or thylakoid configurations (Fig. S7). This suggests that destacking related expansion along the minor axis is generally restricted, consistent with the strong interconnectedness of the thylakoid network, and further masked by the large variability in thylakoid architecture. The nexus zones described here have structural similarities to the convergence zones observed in the thylakoids of some cyanobacteria (Rast et al 2015, 2019). Although the convergence zones are suggested to be involved in thylakoid biogenesis (Stengel et al 2012; Rast et al 2015), further research is needed to assess the function of the nexus zones in plant thylakoids.

In this work, we have shown that the fluorescence characteristics of thylakoid membranes are dependent on the Mg^2+^ concentration, regardless of their prior configuration (stacked or destacked). Our findings support the idea that restacking involves architectural rearrangements of the thylakoid that affect energy transfer between PSII and PSI. The combination of structural and spectroscopic data suggests that Mg^2+^-mediated regulation of membrane architecture could serve as an additional layer of regulation of the light-harvesting reactions in vivo. Free stromal Mg^2+^ concentrations are known to increase from ∼0.5–1 to ∼2–3 mM upon light acclimation (Portis Jr and Heldt 1976; Ishijima et al 2003), likely caused by Mg^2+^ efflux from the thylakoid lumen (Pottosin and Dobrovinskaya 2015). Though, it should be noted that the total Mg^2+^ concentration in chloroplasts is substantially higher, with 90% of the total Mg^2+^ bound by chlorophyll and chelating compounds in the stromal fluid (Kleczkowski and Igamberdiev 2021). However, these values cannot be directly compared to our in vitro experiments conducted in low-salt buffer. In the chloroplast stroma in vivo, the high macromolecular crowding and presence of soluble proteins likely substantially influence stacking behavior (Kim et al 2005). This implies that stacking is more stable in vivo and that subtle changes in thylakoid ultrastructure could occur at lower Mg^2+^ concentrations than in the in vitro experiments.

Cation-dependent changes in the thylakoid organization may be physiologically relevant. Partial grana destacking has been observed in vivo under high light conditions (Kirchhoff et al 2011; Tsabari et al 2015; Li et al 2020), and a recent report has also noted that grana are more tightly packed in the early stages of light acclimation (Wójtowicz et al 2025). This partial destacking is thought to facilitate the repair of light-damaged PSII by allowing bulky repair proteins access to the otherwise tightly packed grana and by facilitating lateral diffusion of PSII (Yoshioka-Nishimura et al 2014), a process that takes place on minute timescale (Goral et al 2010). Our results show that the Mg^2+^-dependent thylakoid reorganizations in vitro also take minutes to occur (Fig. 1; Fig. S2). This suggests that lateral diffusion of protein complexes could be the rate-limiting step in achieving a new structural and functional steady state. In addition, earlier studies have shown that LHCII moves more easily from PSII to PSI at lower Mg^2+^ concentrations (Samson and Bruce 1995), indicating that Mg^2+^ could play a role in the modulation of state transitions. Interestingly, Garty et al (2024) reported that grana doublets become more abundant after 2 h of light compared to overnight dark acclimation. This change was lost in the mutant that lacked the LHCII and PSII kinase, suggesting a role for phosphorylation in increasing the membrane repulsion. This organization might be an intermediate between the fibrous and stacked classes we observed, further supporting a link between Mg^2+^/phosphate-dependent structural flexibility and physiological acclimation.

## Materials and methods

### *A. thaliana* growth conditions

Ecotype *Col-0 A. thaliana* plants were grown on soil under an 8 to 16 h light-dark cycle at 60% humidity. During the light period, the light intensity was ∼125 µmol m^−2^ s^−1^ and the temperature was set to 24 °C. During the dark period, the temperature was set to 22 °C.

### Thylakoid membrane isolation

Isolated thylakoids were prepared to examine the thylakoid destacking and restacking in vitro. Thylakoid membrane isolation occurred in the dark on ice as much as possible. Plants were dark-adapted for at least 12 h. Rosettes of three to six 5.5- to 6.5-wk-old *A. thaliana* plants were homogenized in an ice-cooled blender with ice-cold Grinding Buffer (GB; 400 mM D-sorbitol, 10 mM NaHCO_3_, 5 mM MgCl_2_, 20 mM tricine, and 0.1% (w/v) bovine serum albumin [BSA]; pH 7.0) using 3 sub-second pulses. The homogenate was filtered through 4 layers of cheesecloth and centrifuged (2,000 × *g*, 4 °C, 5 min). The pellet was carefully resuspended in GB using a brush, centrifuged again (2,000 × *g*, 4 °C, 5 min) and again carefully resuspended in 1 mL GB. Ten-milliliter hypo-osmotic breaking buffer (5 mM MgCl_2_ and 10 mM HEPES; pH 7.6) was added to remove the chloroplast envelope. After 30 s of gentle agitation, an equal amount of osmoticum (660 mM D-sorbitol, 5 mM MgCl_2_, 10 mM HEPES, and 20 mM KCl; pH 7.6) was added. The thylakoid suspension was centrifuged again (2,000 × *g*, 4 °C, 5 min). The isolated thylakoid membranes were carefully resuspended in GB and kept on ice. A fraction of the sample was diluted in 80% acetone and centrifuged (15,000 × *g*, 5 min). The absorption at 647 nm (A_674 nm_), 664 nm (A_664 nm_), and 720 nm (A_720 nm_) was measured using a Cary 4000 UV-Vis spectrophotometer. The chlorophyll (Chl) concentration ([Chl_a_  _+_  _b_]) was estimated according to the following equation (Porra et al 1989):


$$\left[Chl_{a+b}](\mu g/mL)=17.76\times (A_{647\;nm}-A_{720\;nm})+7.34\times (A_{664\;nm}-A_{720\;nm}\right)$$

### Stepwise thylakoid restacking and destacking

Thylakoid destacking and restacking were performed using a similar protocol to Kiss et al (2008). The thylakoid solution was diluted with GB to a concentration of approximately 340 µg Chl/mL. Thylakoid destacking and restacking were followed by fluorescence analysis using a DUAL-PAM-100 system (Heinz Walz, Germany) under continuous stirring and constant low actinic light (55 µmol m^−2^ s^−1^) at room temperature. Thylakoids were diluted to 10 µg Chl/mL in resuspension buffer (330 mM D-sorbitol, 5 mM KCl, 15 mM HEPES, 0.1% (w/v) BSA; pH 7.6) in the presence of 0.5 mM EDTA and 50 µM DCMU. DCMU was present to close the PSII RCs in the presence of weak light, thereby maximizing the PSII fluorescence. The thylakoids were allowed to destack under these conditions for 15 min. Then, 0.5 mM MgCl_2_ was added to counteract the present EDTA. Subsequent titrations of MgCl_2_ were performed when the fluorescence signal was stable for at least 1 min. MgCl_2_ additions were carried out until further additions did not increase the fluorescence signal further. At specific Mg^2+^ concentrations, a sample of the thylakoids was taken for ExM and 77 K fluorescence emission measurements.

To follow stepwise destacking, thylakoids were diluted to 10 µg Chl/mL in GB in the presence of 50 µM DCMU. The system was allowed to stabilize for 15 min. Additions of EDTA were carried out similarly to the MgCl_2_ additions in the stepwise restacking. At specific EDTA concentrations, a sample was taken for 77 K fluorescence emission.

### Expansion microscopy

Thylakoids in various states (stacked, destacked, (partially) restacked) were fixed and expanded as described in Berentsen et al (2025), with minor adjustments. These experiments were performed in 3 independent experimental runs. In short, thylakoids were fixed in 3% paraformaldehyde and 0.1% glutaraldehyde in the same buffer as the thylakoids were sampled from (instead of the isolation buffer). Samples were then washed with GB and shortly permeabilized on ice using 0.1% Triton X-100 in GB. After washing, samples were post-fixed using 1% acrylamide and 0.7% paraformaldehyde overnight and washed with GB.

Fixed thylakoids were put on ExM gels as described in Berentsen et al (2025). In short, thylakoids were pelleted and resuspended in ExM monomer solution. N, N, N, N′-tetramethyl ethylenediamine (TEMED) and ammonium persulfate (APS) were added to start the gelation. The gelation occurred in a humidified chamber at 37 °C. Fully formed gels were denatured. Gels were then expanded by several washes in ultrapure water. The expansion factor was determined by comparing the size of the pre-expanded and the post-expanded gel using pictures including a ruler for scale. As the gels from a run were made with the same monomer solution, the average expansion factor of the gels was taken as the correction expansion factor (Berentsen et al 2025). Our previous results have shown that at the gel expansion factors presented here (∼5×), thylakoids expand with the same expansion factor as the gel (Bos et al 2024). Small pieces of the expanded gels were washed with phosphate-buffered saline and stained with 0.13 mg/mL ATTO-594 NHS-ester. This dye targets the primary amines in the sample and therefore allows for the visualization of the proteins present in the sample (Nanda and Lorsch 2014). After staining, gels were re-expanded in ultrapure water.

To minimize drift during imaging, gels were placed in a poly-L-lysine-covered µ-Slide 8 Well slide (Ibidi), as described in Berentsen et al (2025). Images of the expanded thylakoids under various states of stacking were recorded using a confocal STELLARIS-WLL system (Leica Microsystems, Germany) equipped with an HC PL APO CS2 86×/1.20 water objective. The preset configurations to detect ATTO-594 in the LAS X software (Leica Microsystems, Germany) for laser wavelength (602 nm) and HyD X2 detector (614 to 829 nm, “counting” mode) were used. A large low-resolution overview image was used to select 15 to 30 random thylakoids per preparation for detailed imaging, with the only selection criterium being an oval shape, suggesting side views of thylakoids. As the microscopy system produces mirrored images, the images presented here are mirrored back.

### EM

Thylakoids at different stages of restacking were also prepared for imaging using EM. This validation experiment was performed once as a qualitative confirmation of the structures observed in ExM. For this, thylakoids were isolated, destacked, and stepwise restacked as described above in buffers without BSA. The concentration during destacking and restacking was increased to ∼30 µg Chl/mL by using the same volume of a stock solution with a higher thylakoid concentration. This increase in thylakoid concentration did not alter the fluorescence characteristics at different Mg^2+^ concentrations (Fig. S8). Thylakoids in various states were fixed in 2.5% glutaraldehyde in the same buffer from which the thylakoids were sampled for 5 d at 4 °C. Samples were then washed twice in the sampling buffer and twice in 0.1 M phosphate/citrate buffer (pH 7.2). Thylakoids were pelleted and embedded in 3% gelatin in the phosphate/citrate buffer. Sections of the gelatin-embedded samples were cut into small pieces and processed similarly to Zou et al (2024). The samples were fixed in 2.5% glutaraldehyde in the phosphate/citrate buffer overnight at RT. Samples were then washed 6 times in the phosphate/citrate buffer and post-fixed in 1% osmium tetroxide in the phosphate/citrate buffer for 1 h at RT. Samples were washed 3 times in ultrapure water and stepwise dehydrated in ethanol (10 min in 10%, 30%, and 50%; overnight in 70%; 10 min in 80%, 90%, 96%, and 100%; and finally 20 min in 100%). Samples were then infiltrated with Spurr resin via a 3-step ethanol:Spurr gradient (30 min each), followed by 3 infiltrations with 100% Spurr (1 h, overnight, and 1 h). Samples were embedded in BEEM capsules filled with Spurr and polymerized in an oven at 70 °C for 8 h. Samples were sectioned using a Leica Ultramicrotome UC7 and stained with 2% uranyl acetate for 10 min. The samples were washed with ultrapure water, stained with 3% CO_2_-free lead citrate for 10 min, and washed with ultrapure water. Images were recorded with a JEOL JEM 1400 transmission electron microscope (120 kV).

### 77 K fluorescence emission

Samples of thylakoids in various states were frozen in N_2_ (l). As the samples were taken from the same bulk preparation (see above), the chlorophyll concentrations were identical across samples within the same run. The 77 K fluorescence spectra of these samples were recorded using an FS5 fluorometer (Edinburgh Instruments, United Kingdom) equipped with an SC-70 liquid nitrogen dewar. The excitation wavelength was set to 480 nm (±1 to 2 nm). Spectra were recorded between 650 and 800 nm, with a dwell time of 0.2 s/nm. Five spectra were recorded for each sample.

### Data analysis

PAM fluorescence traces and 77 K fluorescence emission spectra were analyzed with custom Python (version 3.11) scripts. The PAM fluorescence traces occasionally showed an increase in signal when a sample was taken. As samples were only taken when the signal was deemed stable, the analysis script corrects for this signal increase (Fig. S9).

77 K fluorescence spectra were smoothed with a Savitzky–Golay filter using a 5-point window size and second polynomial order. Smoothed spectra were normalized to the area between 675 and 705 nm. The peaks of PSI and PSII were determined as the maximum value between 720–750 nm and 675–700 nm, respectively.

ExM and EM images were analyzed using FIJI (FIJI Is Just ImageJ) (Schindelin et al 2012). Computer-assisted classification was attempted on the structures in the ExM images. For this, we attempted a principle component analysis on the grayscale images, as well as on binary images and on Fourier-transformed images of thylakoids. However, the structural classes could not be distinguished using these methods. The ExM images were therefore visually classified based on the main structural feature the thylakoids displayed. Both 2D and 3D representations were used to classify the images. 3D images were prepared using the 3D viewer in the LAS X software (Leica Microsystems, Germany).

## Supplementary Material

kiag101_Supplementary_Data

## Data Availability

The data underlying this publication and Python scripts used for data analysis can be accessed at http://doi.org/10.4121/449bac03-b291-4226-8484-944670081432.

## References

[kiag101-B1] Albanese P, Tamara S, Saracco G, Scheltema RA, Pagliano C. 2020. How paired PSII–LHCII supercomplexes mediate the stacking of plant thylakoid membranes unveiled by structural mass-spectrometry. Nat Commun. 11:1361. 10.1038/s41467-020-15184-1. 32170184 PMC7069969

[kiag101-B2] Albertsson P-Å . 2001. A quantitative model of the domain structure of the photosynthetic membrane. Trends Plant Sci. 6:349–354. 10.1016/s1360-1385(01)02021-0. 11495787

[kiag101-B3] Allen JF, Forsberg J. 2001. Molecular recognition in thylakoid structure and function. Trends Plant Sci. 6:317–326. 10.1016/S1360-1385(01)02010-6. 11435171

[kiag101-B4] Anderson JM, Vernon LP. 1967. Digitonin incubation of spinach chloroplasts in tris (hydroxymethyl) methylglycine solutions of varying ionic strengths. Biochim Biophys Acta. 143:363–376. 10.1016/0005-2728(67)90090-4. 4383083

[kiag101-B5] Aro E-M, Virgin I, Andersson B. 1993. Photoinhibition of photosystem II. Inactivation, protein damage and turnover. Biochim Biophys Acta. 1143:113–134. 10.1016/0005-2728(93)90134-2. 8318516

[kiag101-B6] Barber J . 1980a. An explanation for the relationship between salt-induced thylakoid stacking and the chlorophyll fluorescence changes associated with changes in spillover of energy from photosystem II to photosystem I. FEBS Lett. 118:1–10. 10.1016/0014-5793(80)81207-5.

[kiag101-B7] Barber J . 1980b. Membrane surface charges and potentials in relation to photosynthesis. Biochim Biophys Acta. 594:253–308. 10.1016/0304-4173(80)90003-8. 7018576

[kiag101-B8] Barber J . 1982. Influence of surface charges on thylakoid structure and function. Annu Rev Plant Physiol. 33:261–295. 10.1146/annurev.pp.33.060182.001401.

[kiag101-B9] Bassi R, Hinz U, Barbato R. 1985. The role of the light harvesting complex and photosystem II in thylakoid stacking in the chlorina-f2 barley mutant. Carlsberg Res Commun. 50:347–367. 10.1007/BF02907157.

[kiag101-B10] Berentsen J, Bos PR, Wientjes E. 2025. Expansion microscopy reveals thylakoid organisation alterations due to genetic mutations and far-red light acclimation. Biochim Biophys Acta. 1866:149552. 10.1016/j.bbabio.2025.149552. 40086741

[kiag101-B11] Blankenship RE . 2021. Molecular mechanisms of photosynthesis. John Wiley & Sons. 10.1002/9780470758472.

[kiag101-B12] Bos PR, Berentsen J, Wientjes E. 2024. Expansion microscopy resolves the thylakoid structure of spinach. Plant Physiol. 194:347–358. 10.1093/plphys/kiad526. PMC10756755 37792700

[kiag101-B13] Briantais J-M, Vernotte C, Olive J, Wollman F-A. 1984. Kinetics of cation-induced changes of photosystem II fluorescence and of lateral distribution of the two photosystems in the thylakoid membranes of pea chloroplasts. Biochim Biophys Acta. 766:1–8. 10.1016/0005-2728(84)90210-X.

[kiag101-B14] Burke JJ, Steinback KE, Arntzen CJ. 1979. Analysis of the light-harvesting pigment-protein complex of wild type and a chlorophyll-b-less mutant of barley. Plant Physiol. 63:237–243. 10.1104/pp.63.2.237. 16660704 PMC542805

[kiag101-B15] Bussi Y et al 2019. Fundamental helical geometry consolidates the plant photosynthetic membrane. Proc Natl Acad Sci U S A. 116:22366–22375. 10.1073/pnas.1905994116. 31611387 PMC6825288

[kiag101-B16] Carter DP, Staehelin LA. 1980. Proteolysis of chloroplast thylakoid membranes. II. Evidence for the involvement of the light-harvesting chlorophyll ab-protein complex in thylakoid stacking and for effects of magnesium ions on photosystem II-light-harvesting complex aggregates in the absence of membrane stacking. Arch Biochem Biophys. 200:374–386. 10.1016/0003-9861(80)90367-7. 7002041

[kiag101-B17] Chen F, Tillberg PW, Boyden ES. 2015. Expansion microscopy. Science. 347:543–548. 10.1126/science.1260088. 25592419 PMC4312537

[kiag101-B18] Chow WS, Kim E-H, Horton P, Anderson JM. 2005. Granal stacking of thylakoid membranes in higher plant chloroplasts: the physicochemical forces at work and the functional consequences that ensue. Photochem Photobiol Sci. 4:1081–1090. 10.1039/B507310N. 16307126

[kiag101-B19] Fristedt R, Granath P, Vener AV. 2010. A protein phosphorylation threshold for functional stacking of plant photosynthetic membranes. PLoS One. 5:e10963. 10.1371/journal.pone.0010963. 20532038 PMC2881038

[kiag101-B20] Garty Y et al 2024. Thylakoid membrane stacking controls electron transport mode during the dark-to-light transition by adjusting the distances between PSI and PSII. Nat Plants. 10:512–524. 10.1038/s41477-024-01628-9. 38396112

[kiag101-B21] Goral TK et al 2010. Visualizing the mobility and distribution of chlorophyll proteins in higher plant thylakoid membranes: effects of photoinhibition and protein phosphorylation. Plant J. 62:948–959. 10.1111/j.1365-313X.2010.04207.x. 20230505

[kiag101-B22] Henzl MT, Larson JD, Agah S. 2003. Estimation of parvalbumin Ca2+-and Mg2+-binding constants by global least-squares analysis of isothermal titration calorimetry data. Anal Biochem. 319:216–233. 10.1016/S0003-2697(03)00288-4. 12871715

[kiag101-B23] Herbstová M, Tietz S, Kinzel C, Turkina MV, Kirchhoff H. 2012. Architectural switch in plant photosynthetic membranes induced by light stress. Proc Natl Acad Sci U S A. 109:20130–20135. 10.1073/pnas.1214265109. 23169624 PMC3523818

[kiag101-B24] Hodges M, Briantais J-M, Moya I. 1987. The effect of thylakoid membrane reorganisation on chlorophyll fluorescence lifetime components: a comparison between state transitions, protein phosphorylation and the absence of Mg2+. Biochim Biophys Acta. 893:480–489. 10.1016/0005-2728(87)90099-5.

[kiag101-B25] Isaakidou J, Papageorgiou G. 1975. A fluorimetric study of Mg2+-induced structural changes in thylakoid membrane protein. Arch Biochem Biophys. 168:266–272. 10.1016/0003-9861(75)90250-7. 1137397

[kiag101-B26] Ishijima S, Uchibori A, Takagi H, Maki R, Ohnishi M. 2003. Light-induced increase in free Mg2+ concentration in spinach chloroplasts: measurement of free Mg2+ by using a fluorescent probe and necessity of stromal alkalinization. Arch Biochem Biophys. 412:126–132. 10.1016/S0003-9861(03)00038-9. 12646275

[kiag101-B27] Itoh S, Sugiura K. 2004. Fluorescence of photosystem I. In: Papageorgiou GC, G ovindjee, editors. Chlorophyll a fluorescence: a signature of photosynthesis. Springer. p. 231–250. 10.1007/978-1-4020-3218-9_9.

[kiag101-B28] Izawa S, Good NE. 1966. Effect of salts and electron transport on the conformation of isolated chloroplasts. II. Electron microscopy. Plant Physiol. 41:544–552. 10.1104/pp.41.3.544. 16656286 PMC1086379

[kiag101-B29] Jennings RC, Forti G, Gerola PD, Garlaschi FM. 1978. Studies on cation-induced thylakoid membrane stacking, fluorescence yield, and photochemical efficiency. Plant Physiol. 62:879–884. 10.1104/pp.62.6.879. 16660630 PMC1092246

[kiag101-B30] Johnson MP, Wientjes E. 2020. The relevance of dynamic thylakoid organisation to photosynthetic regulation. Biochim Biophys Acta. 1861:148039. 10.1016/j.bbabio.2019.06.011. 31228404

[kiag101-B31] Kaňa R, Govindjee. 2016. Role of ions in the regulation of light-harvesting. Front Plant Sci. 7:1849. 10.3389/fpls.2016.01849. 28018387 PMC5160696

[kiag101-B32] Kansy M, Wilhelm C, Goss R. 2014. Influence of thylakoid membrane lipids on the structure and function of the plant photosystem II core complex. Planta. 240:781–796. 10.1007/s00425-014-2130-2. 25063517

[kiag101-B33] Kim E-H, Chow WS, Horton P, Anderson JM. 2005. Entropy-assisted stacking of thylakoid membranes. Biochim Biophys Acta. 1708:187–195. 10.1016/j.bbabio.2005.03.011. 15953475

[kiag101-B34] Kirchhoff H et al 2007. Structural and functional self-organization of Photosystem II in grana thylakoids. Biochim Biophys Acta. 1767:1180–1188. 10.1016/j.bbabio.2007.05.009. 17617373

[kiag101-B35] Kirchhoff H et al 2011. Dynamic control of protein diffusion within the granal thylakoid lumen. Proc Natl Acad Sci U S A. 108:20248–20253. 10.1073/pnas.1104141109. 22128333 PMC3250138

[kiag101-B36] Kirchhoff H . 2013. Architectural switches in plant thylakoid membranes. Photosynth Res. 116:481–487. 10.1007/s11120-013-9843-0. 23677426

[kiag101-B37] Kirchhoff H . 2014. Diffusion of molecules and macromolecules in thylakoid membranes. Biochim Biophys Acta. 1837:495–502. 10.1016/j.bbabio.2013.11.003. 24246635

[kiag101-B38] Kisielowski C et al 2008. Detection of single atoms and buried defects in three dimensions by aberration-corrected electron microscope with 0.5-Å information limit. Microsc Microanal. 14:469–477. 10.1017/S1431927608080902. 18793491

[kiag101-B39] Kiss AZ, Ruban AV, Horton P. 2008. The PsbS protein controls the organization of the photosystem II antenna in higher plant thylakoid membranes. J Biol Chem. 283:3972–3978. 10.1074/jbc.M707410200. 18055452

[kiag101-B40] Kleczkowski LA, Igamberdiev AU. 2021. Magnesium signaling in plants. Int J Mol Sci. 22:1159. 10.3390/ijms22031159. 33503839 PMC7865908

[kiag101-B41] Krause GH, Briantais J-M, Vernotte C. 1983. Characterization of chlorophyll fluorescence quenching in chloroplasts by fluorescence spectroscopy at 77 K I. ΔpH-dependent quenching. Biochim Biophys Acta. 723:169–175. 10.1016/0005-2728(83)90116-0.

[kiag101-B42] Krause GH, Weis E. 1991. Chlorophyll fluorescence and photosynthesis: the basics. Annu Rev Plant Biol. 42:313–349. 10.1146/annurev.pp.42.060191.001525.

[kiag101-B43] Kunz H-H, Armbruster U, Mühlbauer S, de Vries J, Davis GA. 2024. Chloroplast ion homeostasis–what do we know and where should we go? New Phytol. 243:543–559. 10.1111/nph.19661. 38515227

[kiag101-B44] Lemeille S, Rochaix J-D. 2010. State transitions at the crossroad of thylakoid signalling pathways. Photosynth Res. 106:33–46. 10.1007/s11120-010-9538-8. 20217232

[kiag101-B45] Li M et al 2020. Measuring the dynamic response of the thylakoid architecture in plant leaves by electron microscopy. Plant Direct. 4:e00280. 10.1002/pld3.280. 33195966 PMC7644818

[kiag101-B46] Messant M, Krieger-Liszkay A, Shimakawa G. 2021. Dynamic changes in protein-membrane association for regulating photosynthetic electron transport. Cells. 10:1216. 10.3390/cells10051216. 34065690 PMC8155901

[kiag101-B47] Nakatani H, Barber J, Forrester J. 1978. Surface charges on chloroplast membranes as studied by particle electrophoresis. Biochim Biophys Acta. 504:215–225. 10.1016/0005-2728(78)90019-1. 30479

[kiag101-B48] Nanda JS, Lorsch JR. 2014. Labeling a protein with fluorophores using NHS ester derivitization. Methods Enzymol. 536:87–94. 10.1016/b978-0-12-420070-8.00008-8. 24423269

[kiag101-B49] Nevo R et al 2009. Architecture of thylakoid membrane networks. In: Wada H, Murata N, editors. Lipids in photosynthesis: essential and regulatory functions. Springer. p. 295–328. 10.1007/978-90-481-2863-1_14.

[kiag101-B50] Nevo R, Charuvi D, Tsabari O, Reich Z. 2012. Composition, architecture and dynamics of the photosynthetic apparatus in higher plants. Plant J. 70:157–176. 10.1111/j.1365-313x.2011.04876.x. 22449050

[kiag101-B51] O’Brien LC et al 2015. M2+• EDTA binding affinities: a modern experiment in thermodynamics for the physical chemistry laboratory. J Chem Educ. 92:1547–1551. 10.1021/acs.jchemed.5b00159.

[kiag101-B52] Paolillo Jr DJ . 1970. The three-dimensional arrangement of intergranal lamellae in chloroplasts. J Cell Sci. 6:243–253. 10.1242/jcs.6.1.243. 5417695

[kiag101-B53] Perez-Boerema A, Engel BD, Wietrzynski W. 2024. Evolution of thylakoid structural diversity. Annu Rev Cell Dev Biol. 40:169–193. 10.1146/annurev-cellbio-120823-022747. 38950450

[kiag101-B54] Porra RJ, Thompson WA, Kriedemann PE. 1989. Determination of accurate extinction coefficients and simultaneous equations for assaying chlorophylls a and b extracted with four different solvents: verification of the concentration of chlorophyll standards by atomic absorption spectroscopy. Biochim Biophys Acta. 975:384–394. 10.1016/S0005-2728(89)80347-0.

[kiag101-B55] Portis Jr AR, Heldt HW. 1976. Light-dependent changes of the Mg2+ concentration in the stroma in relation to the Mg2+ dependency of CO2 fixation in intact chloroplasts. Biochim Biophys Acta. 449:434–446. 10.1016/0005-2728(76)90154-7. 11816

[kiag101-B56] Pottosin I, Dobrovinskaya O. 2015. Ion channels in native chloroplast membranes: challenges and potential for direct patch-clamp studies. Front Physiol. 6:396. 10.3389/fphys.2015.00396. 26733887 PMC4686732

[kiag101-B57] Pribil M, Labs M, Leister D. 2014. Structure and dynamics of thylakoids in land plants. J Exp Bot. 65:1955–1972. 10.1093/jxb/eru090. 24622954

[kiag101-B58] Puthiyaveetil S et al 2014. Compartmentalization of the protein repair machinery in photosynthetic membranes. Proc Natl Acad Sci U S A. 111:15839–15844. 10.1073/pnas.1413739111. 25331882 PMC4226077

[kiag101-B59] Puthiyaveetil S, Van Oort B, Kirchhoff H. 2017. Surface charge dynamics in photosynthetic membranes and the structural consequences. Nat Plants. 3:1–9. 10.1038/nplants.2017.20. 28263304

[kiag101-B60] Rantala M, Rantala S, Aro E-M. 2020. Composition, phosphorylation and dynamic organization of photosynthetic protein complexes in plant thylakoid membrane. Photochem Photobiol Sci. 19:604–619. 10.1039/d0pp00025f. 32297616

[kiag101-B61] Rast A et al 2019. Biogenic regions of cyanobacterial thylakoids form contact sites with the plasma membrane. Nat Plants. 5:436–446. 10.1038/s41477-019-0399-7. 30962530

[kiag101-B62] Rast A, Heinz S, Nickelsen J. 2015. Biogenesis of thylakoid membranes. Biochim Biophys Acta. 1847:821–830. 10.1016/j.bbabio.2015.01.007. 25615584

[kiag101-B63] Rojdestvenski I et al 2002. Segregation of photosystems in thylakoid membranes as a critical phenomenon. Biophys J. 82:1719–1730. 10.1016/S0006-3495(02)75524-0. 11916833 PMC1301971

[kiag101-B64] Rumak I et al 2010. 3-D modelling of chloroplast structure under (Mg2+) magnesium ion treatment. Relationship between thylakoid membrane arrangement and stacking. Biochim Biophys Acta. 1797:1736–1748. 10.1016/j.bbabio.2010.07.001. 20621057

[kiag101-B65] Samson G, Bruce D. 1995. Complementary changes in absorption cross-sections of photosystems I and II due to phosphorylation and Mg2+-depletion in spinach thylakoids. Biochim Biophys Acta. 1232:21–26. 10.1016/0005-2728(95)00104-6.

[kiag101-B66] Schindelin J et al 2012. Fiji: an open-source platform for biological-image analysis. Nat Methods. 9:676–682. 10.1038/nmeth.2019. 22743772 PMC3855844

[kiag101-B67] Staehelin LA . 1986. Chloroplast structure and supramolecular organization of photosynthetic membranes. In: Staehelin LA, Arntzen CJ, editors. Photosynthesis III: photosynthetic membranes and light harvesting systems. Springer. p. 1–84. 10.1007/978-3-642-70936-4_1.

[kiag101-B68] Staehelin LA, van der Staay GW. 1996. Structure, composition, functional organization and dynamic properties of thylakoid membranes. In: Ort DR, Yocum CF, Heichel IF, editors. Oxygenic photosynthesis: the light reactions. Springer. p. 11–30. 10.1007/0-306-48127-8_2.

[kiag101-B69] Standfuss J, Terwisscha van Scheltinga AC, Lamborghini M, Kühlbrandt W. 2005. Mechanisms of photoprotection and nonphotochemical quenching in pea light-harvesting complex at 2.5 Å resolution. EMBO J. 24:919–928. 10.1038/sj.emboj.7600585. 15719016 PMC554132

[kiag101-B70] Stengel A et al 2012. Initial steps of photosystem II de novo assembly and preloading with manganese take place in biogenesis centers in Synechocystis. Plant Cell. 24:660–675. 10.1105/tpc.111.093914. 22319052 PMC3315239

[kiag101-B71] Tikkanen M et al 2006. State transitions revisited—a buffering system for dynamic low light acclimation of Arabidopsis. Plant Mol Biol. 62:779–793. 10.1007/s11103-006-9044-8. 16897465

[kiag101-B72] Tikkanen M, Grieco M, Aro E-M. 2011. Novel insights into plant light-harvesting complex II phosphorylation and ‘state transitions’. Trends Plant Sci. 16:126–131. 10.1016/j.tplants.2010.11.006. 21183394

[kiag101-B73] Trissl H-W, Wilhelm C. 1993. Why do thylakoid membranes from higher plants form grana stacks? Trends Biochem Sci. 18:415–419. 10.1016/0968-0004(93)90136-B. 8291084

[kiag101-B74] Trotta A et al 2025. Defining the heterogeneous composition of Arabidopsis thylakoid membrane. Plant J. 121:e17259. 10.1111/tpj.17259. 39930594 PMC11811488

[kiag101-B75] Tsabari O et al 2015. Differential effects of ambient or diminished CO 2 and O2 levels on thylakoid membrane structure in light-stressed plants. Plant J. 81:884–894. 10.1111/tpj.12774. 25619921

[kiag101-B76] Van der Weij-de Wit C, Ihalainen J, Van Grondelle R, Dekker J. 2007. Excitation energy transfer in native and unstacked thylakoid membranes studied by low temperature and ultrafast fluorescence spectroscopy. Photosynth Res. 93:173–182. 10.1007/s11120-007-9157-1. 17390231

[kiag101-B77] Wan T et al 2014. Crystal structure of a multilayer packed major light-harvesting complex: implications for grana stacking in higher plants. Mol Plant. 7:916–919. 10.1093/mp/ssu005. 24482437

[kiag101-B78] Wietrzynski W et al 2025. Molecular architecture of thylakoid membranes within intact spinach chloroplasts. Elife. 14:RP105496. 10.7554/eLife.105496.3. PMC12425480 40932103

[kiag101-B79] Wójtowicz J et al 2025. Shrink or expand? Just relax! Bidirectional grana structural dynamics as early light-induced regulator of photosynthesis. New Phytol. 246:2580–2596. 10.1111/nph.70175. 40289507 PMC12095992

[kiag101-B80] Wood WH et al 2018. Dynamic thylakoid stacking regulates the balance between linear and cyclic photosynthetic electron transfer. Nat Plants. 4:116–127. 10.1038/s41477-017-0092-7. 29379151

[kiag101-B81] Wood WH, Barnett SF, Flannery S, Hunter CN, Johnson MP. 2019. Dynamic thylakoid stacking is regulated by LHCII phosphorylation but not its interaction with PSI. Plant Physiol. 180:2152–2166. 10.1104/pp.19.00503.

[kiag101-B82] Yoshioka-Nishimura M et al 2014. Quality control of photosystem II: direct imaging of the changes in the thylakoid structure and distribution of FtsH proteases in spinach chloroplasts under light stress. Plant Cell Physiol. 55:1255–1265. 10.1093/pcp/pcu079. 24891560

[kiag101-B83] Zou Y et al 2024. Arabinosylation of cell wall extensin is required for the directional response to salinity in roots. Plant Cell. 36:3328–3343. 10.1093/plcell/koae135. 38691576 PMC11371136

