# Supplementary material for: Ionic regulation of thylakoid membrane architecture: Mg2+-driven destacking and restacking visualized
Source: Plant Physiol. 2026 Feb 27;200(4):kiag101. doi: 10.1093/plphys/kiag101 (PMC13070708; doi:10.1093/plphys/kiag101)
Supplement: kiag101_Supplementary_Data [file kiag101_supplementary_data.pdf]

# Supplementary Information: Ionic regulation of thylakoid membrane architecture: $Mg^{2+}$-driven destacking and restacking visualized

Jarne Berentsen[1], Erwin Hogeveen[1], Emilie Wientjes[1*]

[1]Laboratory of Biophysics, Wageningen University & Research, 6708 WE, Wageningen, the Netherlands

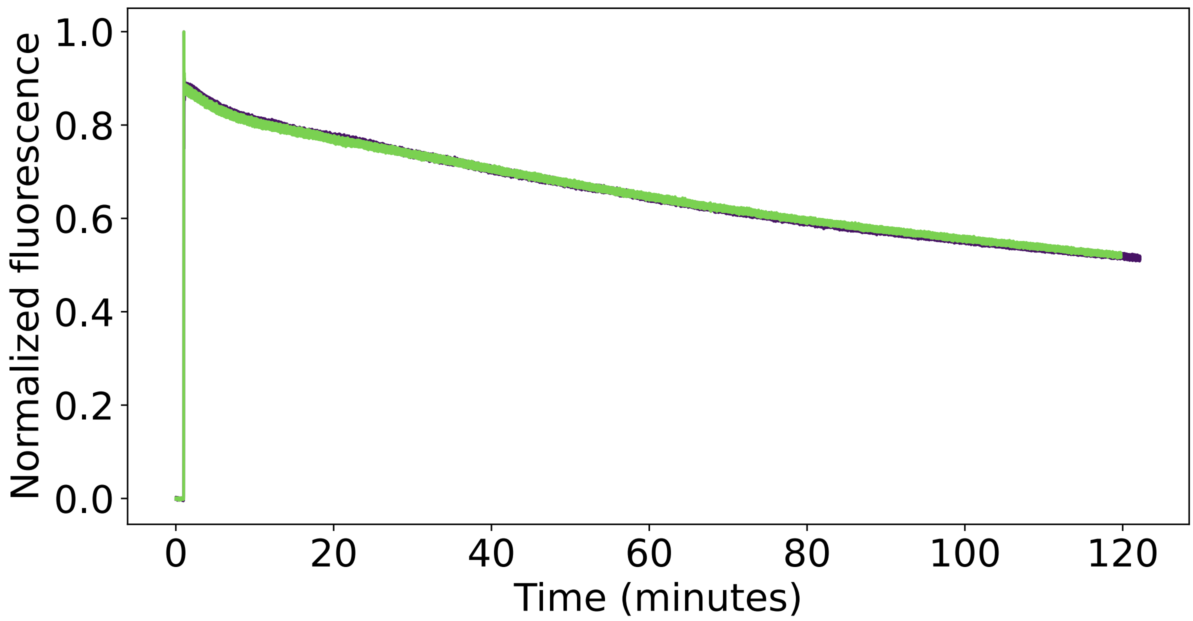


**Figure S1.** Decrease in $F_m$ of stacked thylakoids due to photoinhibition caused by the experimental settings. Stacked thylakoids were added at minute 1 to the isolation buffer (with sufficient $Mg^{2+}$) with 50 μM DCMU and measured using the DUAL-PAM system in the same settings as described in the main article. These conditions include continuous stirring at room temperature and constant actinic light of 55 μmol $m^{-2}$ $s^{-1}$. Under these conditions, $F_m$ is measured, and the experimental conditions result in a decrease in fluorescence signal.

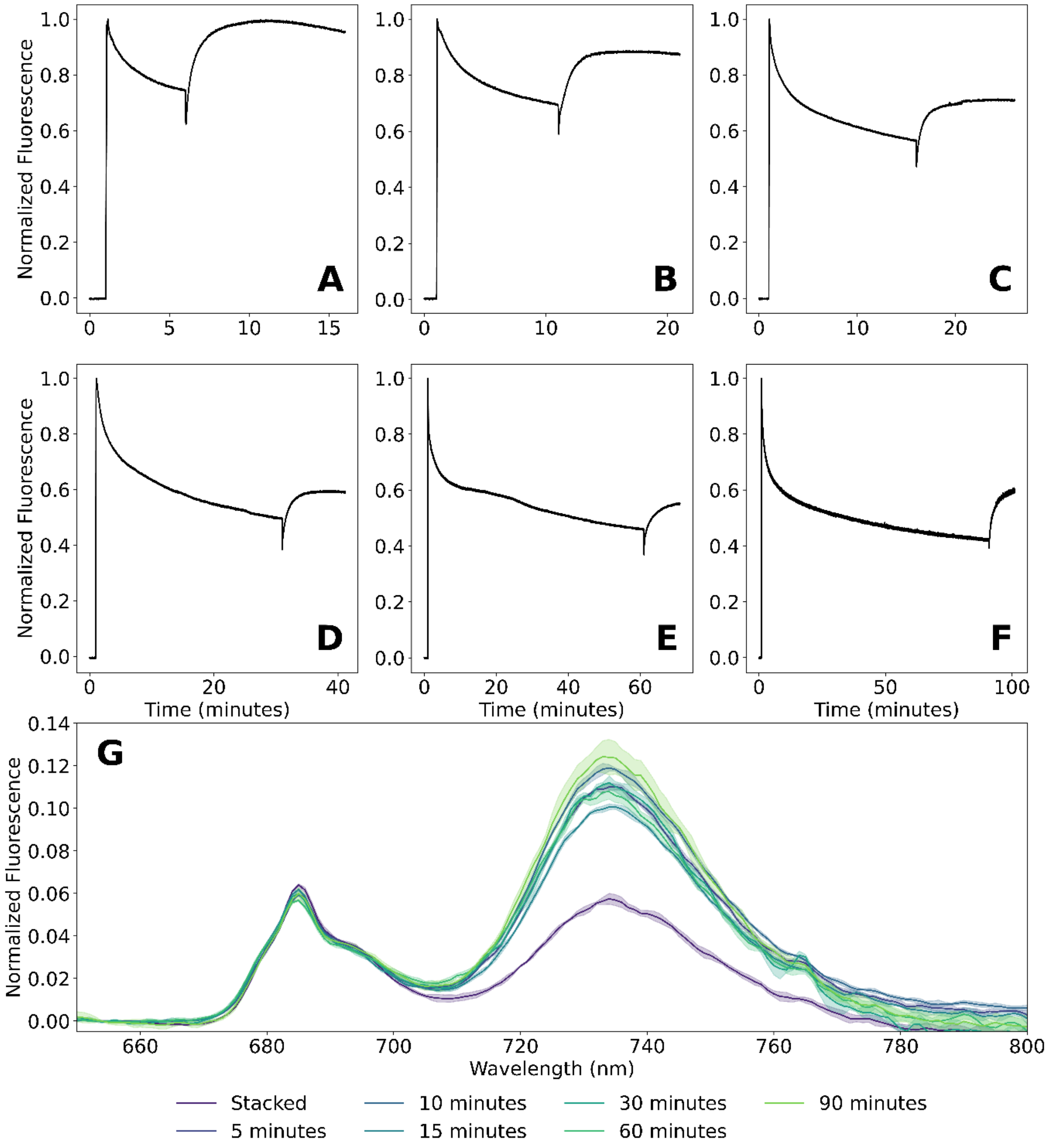


**Figure S2.** Thylakoid destacking in the presence of an excess of EDTA for different durations. Thylakoid destacking in the presence of an excess of EDTA for (A) 5 minutes, (B) 10 minutes, (C) 15 minutes, (D) 30 minutes, (E) 60 minutes, or (F) 90 minutes. After this time, an excess of $MgCl_2$ was added to investigate whether restacking was possible. The restacking was allowed to continue for 10 minutes after addition of the $MgCl_2$. (G) 77K fluorescence emission spectra of thylakoids after various durations of destacking, normalised to area under the PSII peak (675-705 nm).

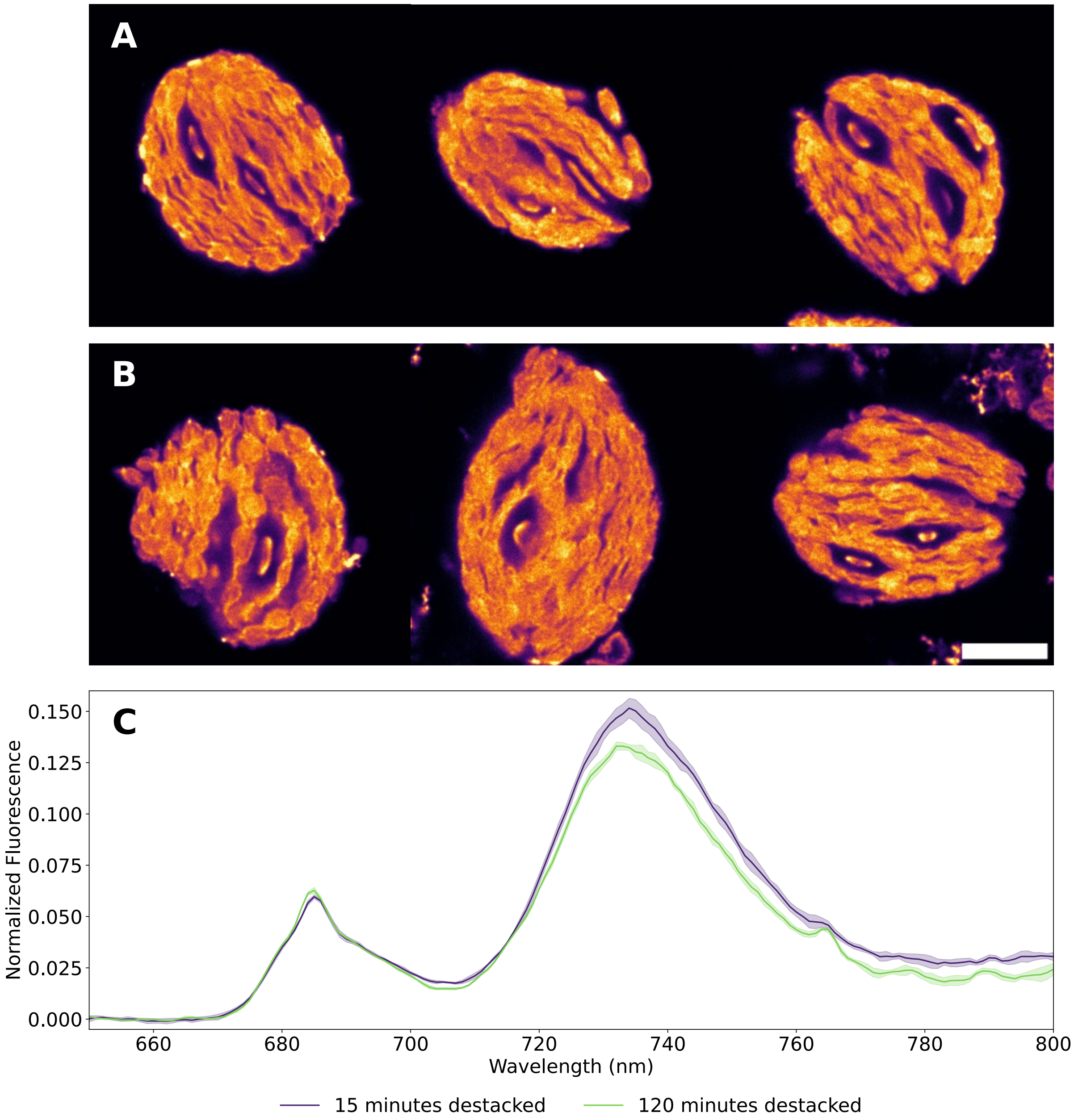


**Figure S3.** ExM images and 77K fluorescence emission of thylakoids destacked for 15 or 120 minutes. (A) ExM examples of thylakoids destacked for (A) 15 minutes or (B) 120 minutes in an excess of EDTA. Scale bar represents 2 μm (corrected for the ~4.5× expansion) and is applicable to all micrographs. (C) 77K fluorescence emission spectra of thylakoids destacked for either 15 or 120 minutes.

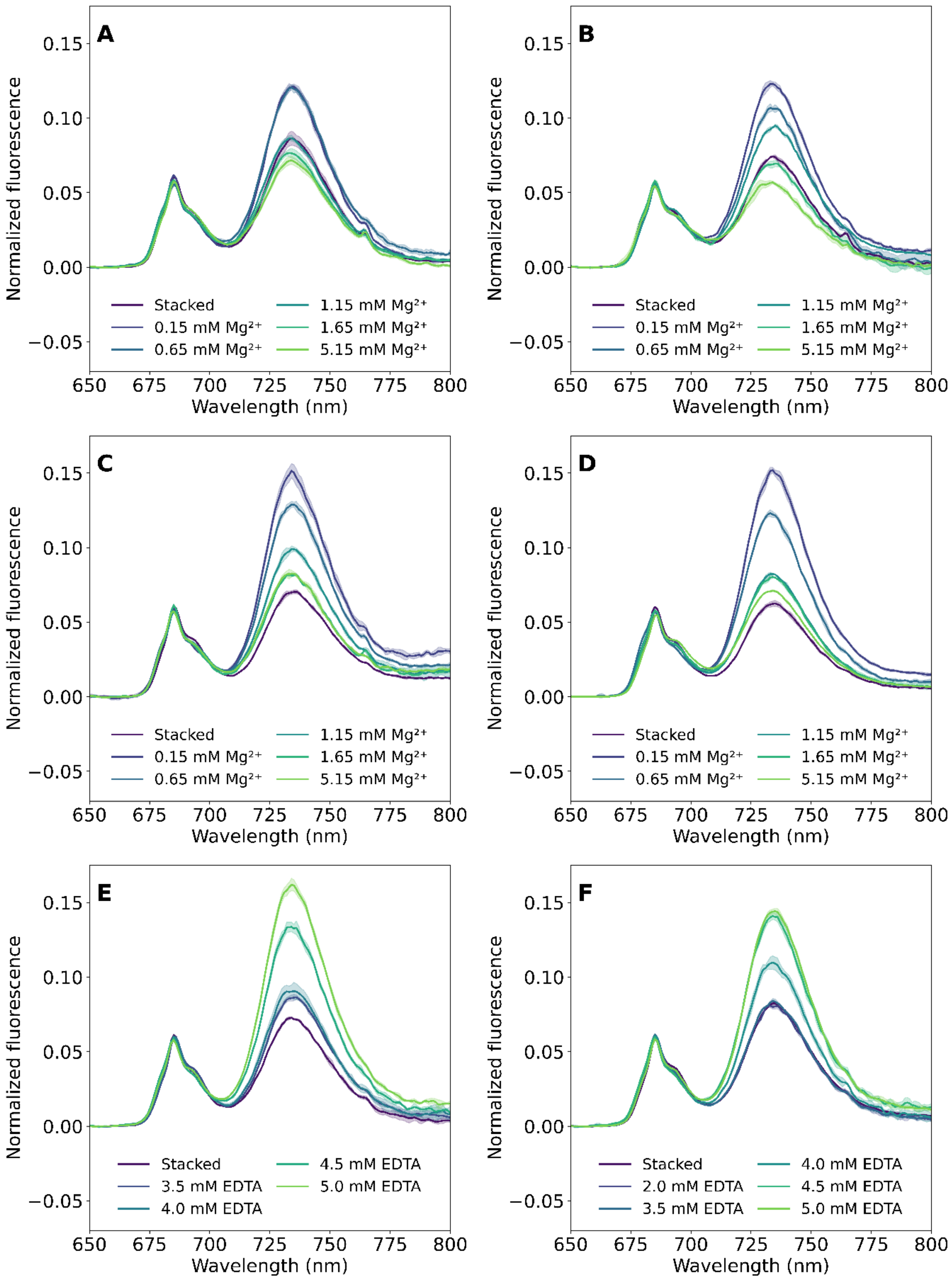


**Figure S4.** Reproducibility of 77K fluorescence traces. Reproducibility of 77K fluorescence traces upon (A-D) stepwise restacking with $MgCl_2$ titrations and (E,F) stepwise destacking with EDTA titrations. (C) and (F) are also presented as Figure 2A and C, respectively.

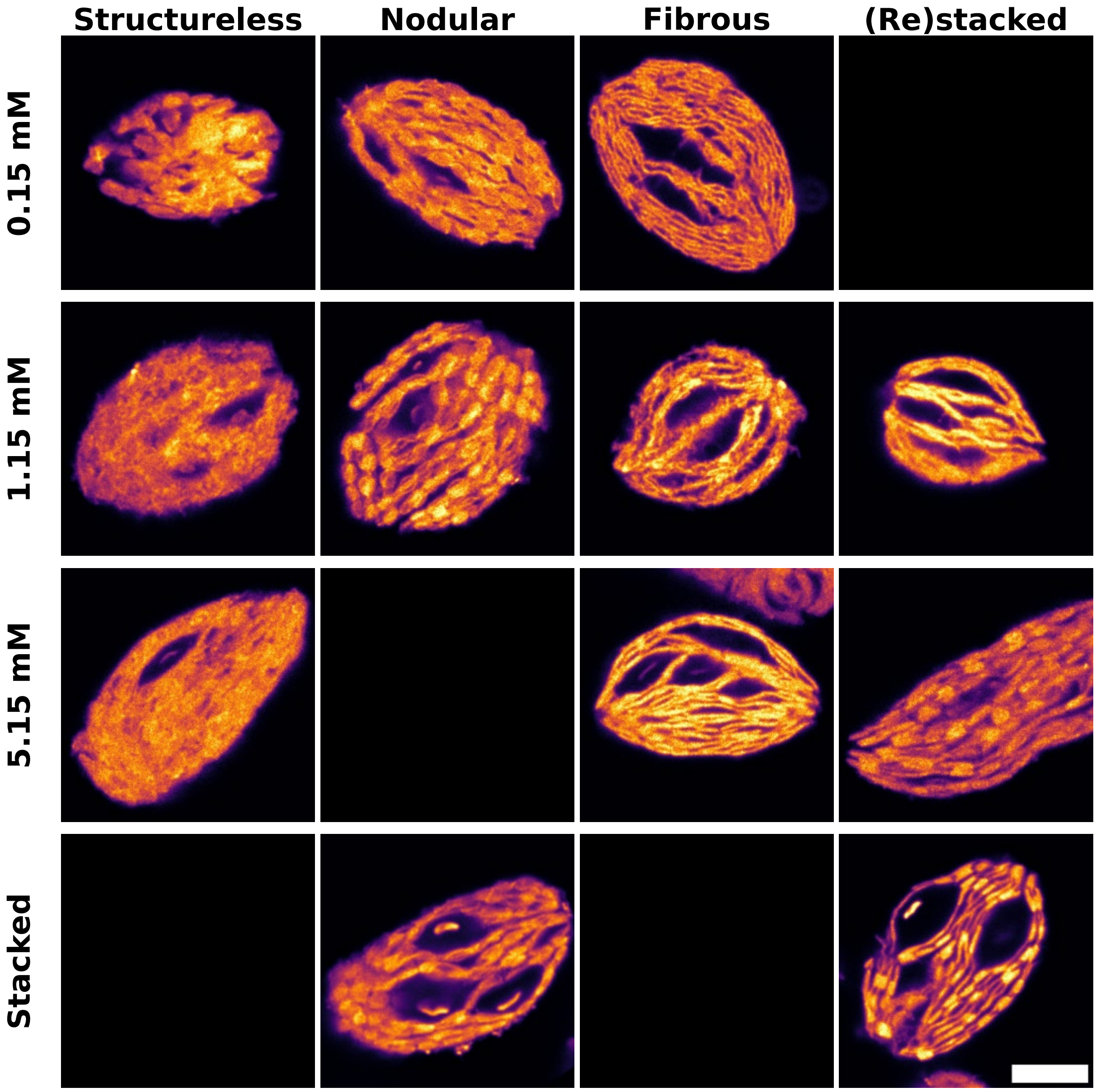


**Figure S5:** Zoomed-out examples of thylakoid ExM images at different $Mg^{2+}$ concentrations. Often, zoomed out examples of the different classes per $Mg^{2+}$ concentration as presented as Figure 4A are shown. Scale bar represents 2 µm, corrected for the expansion factor (~4.6×) and is applicable to all presented examples. Samples were stained with ATTO-594 NHS ester for all-protein visualisation. The absence of an image indicates that class was not found at the $Mg^{2+}$ concentration.

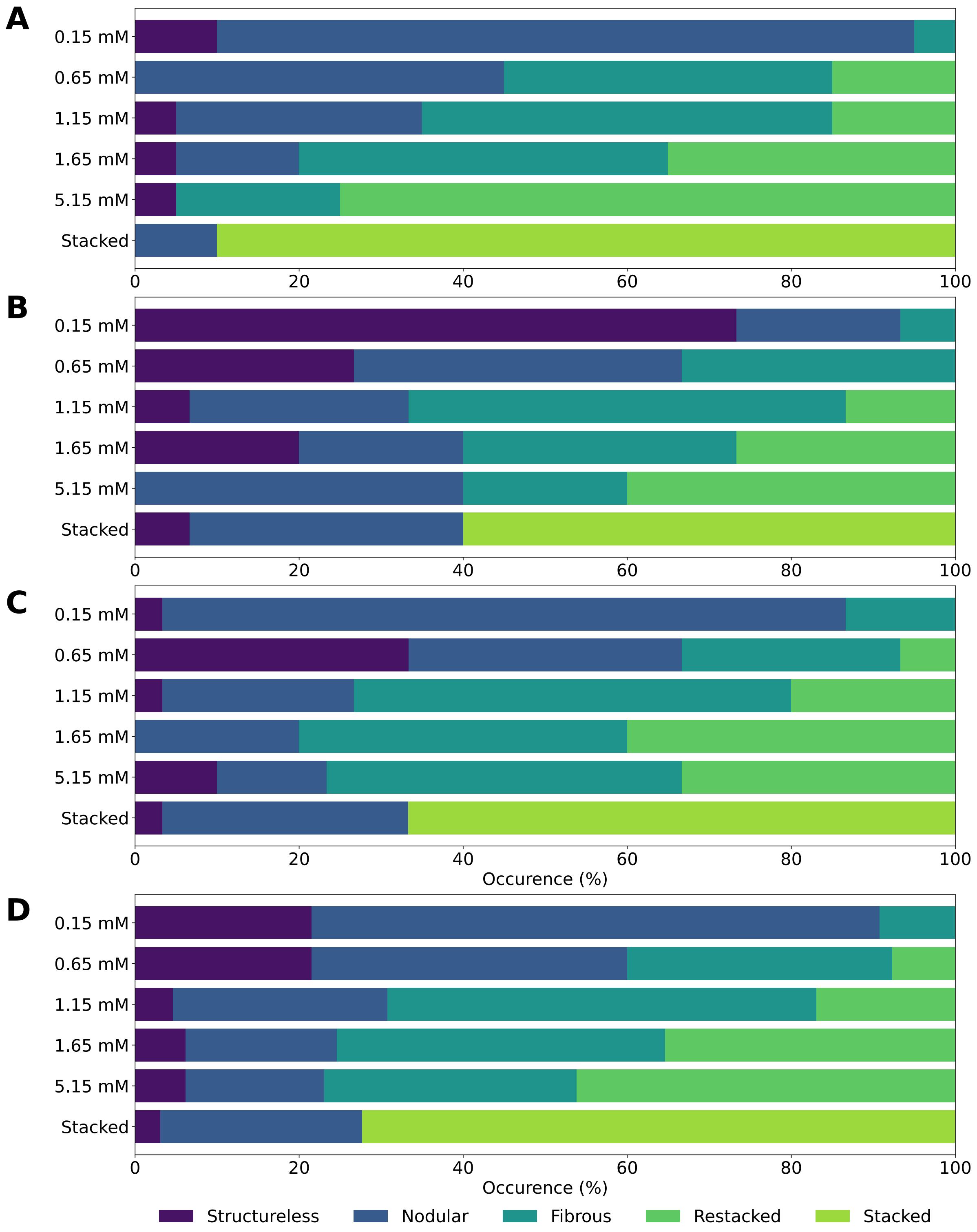


**Figure S6.** Distribution of the five thylakoid structural classes at different $Mg^{2+}$ concentrations. (A-C) Distribution of the five thylakoid structural classes of three independent sample preparations. (D) Total distribution of classifications pooled from the three independent sample preparations. (A) is also presented as Figure 4B. In all cases, restacking appears to happen in two phases. In the first phase, thylakoids move from a nodular to a fibrous architecture. In the second phase, the fibrous architecture is folded to form the grana again, resulting in the restacked architecture.

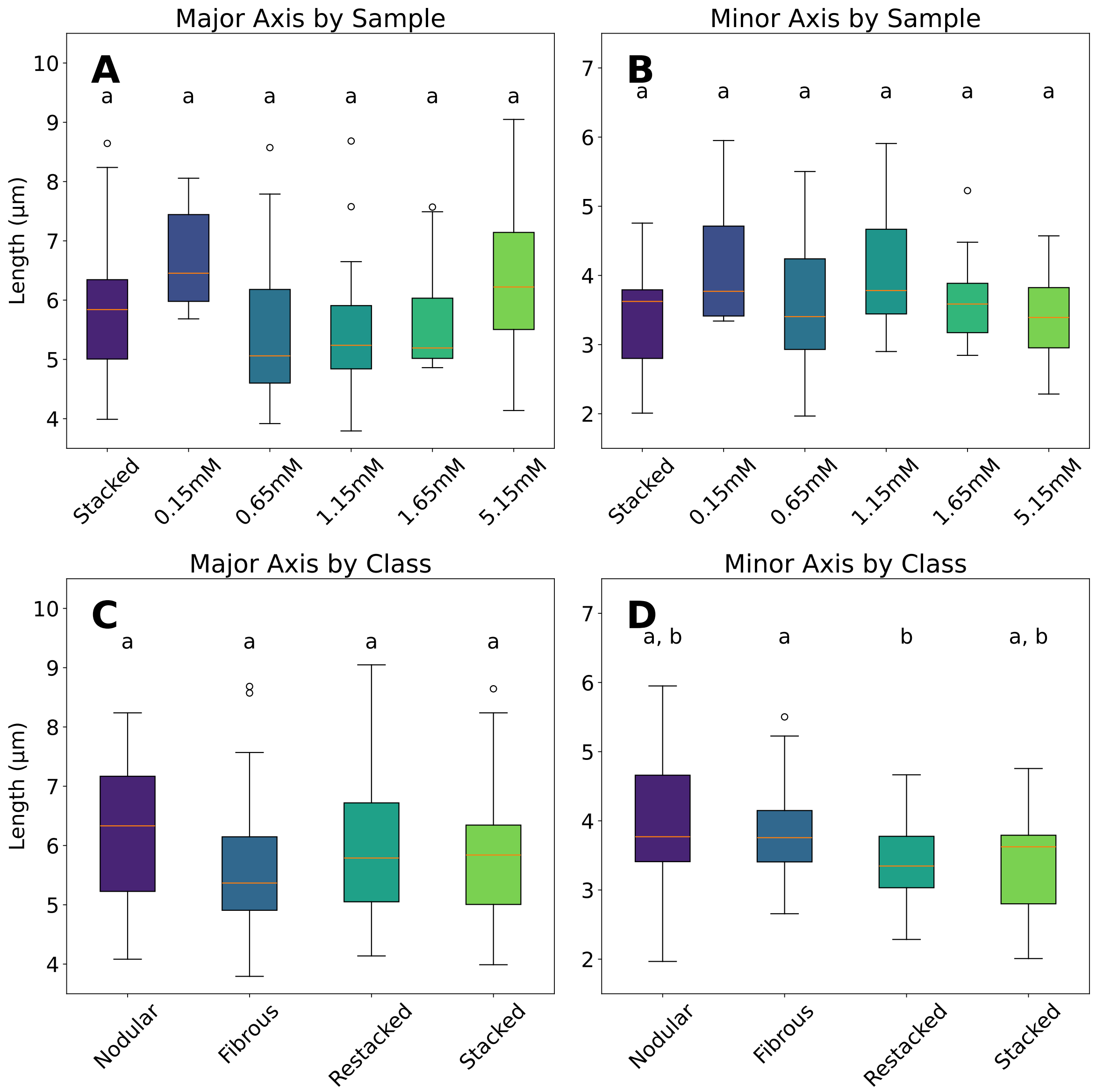


**Figure S7.** Major and minor axis assessment per $Mg^{2+}$ concentration and per class. (A) Major axis length of thylakoids at different $Mg^{2+}$ concentration. (B) Minor axis length of thylakoids at different $Mg^{2+}$ concentration. (C) Major axis length of thylakoids per class. (D) Minor axis length of thylakoid per class. Lengths are corrected for the expansion factor (~4.6×). In the data analysis, the major axis was defined as the axis parallel to the convergence zones and the minor axis as the axis perpendicular to the convergence zones. Letters indicate statistically different groups ($p < 0.05$) using the Kruskal-Wallis test, complemented with Dunn's test with Bonferroni correction. In the presented boxplots, the red line signifies the median. The box spans from the first quartile (Q1) to the third quartile (Q3), representing the interquartile range (IQR). The whiskers extend to the most extreme data points within 1.5 × IQR of the quartiles. Observations beyond this range are plotted individually as outliers.

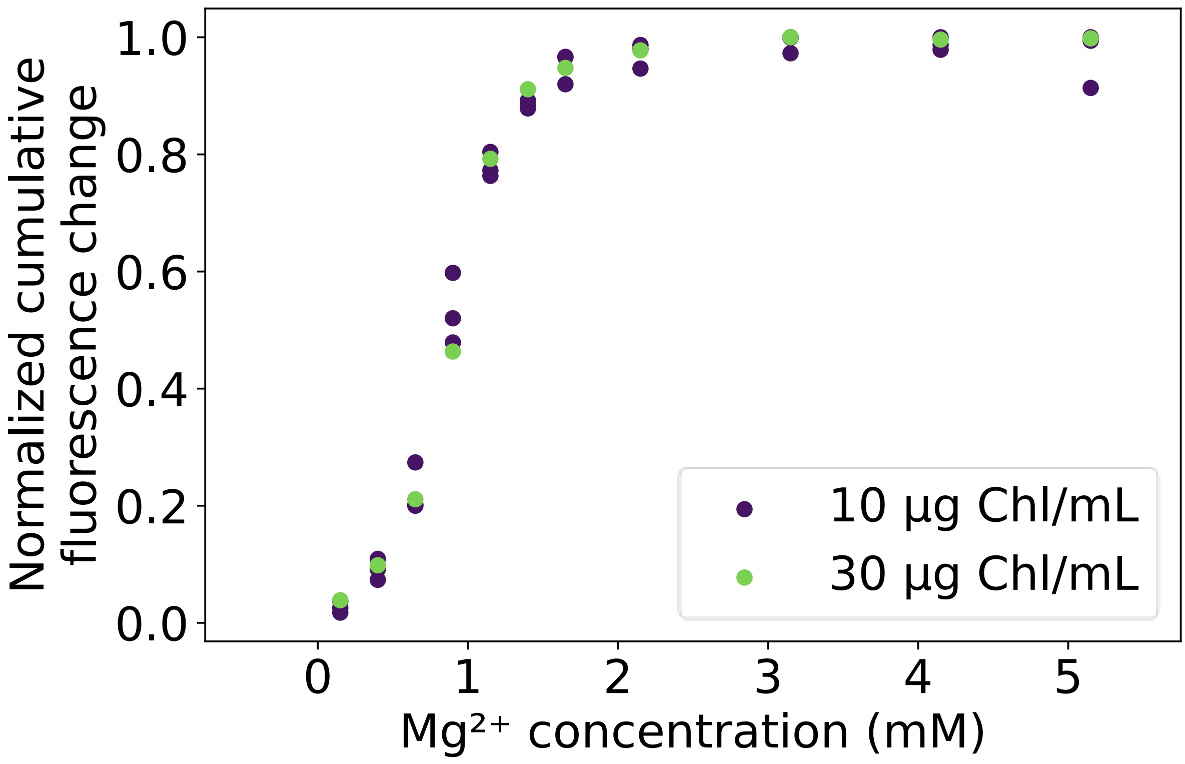


**Figure S8.** Cumulative fluorescence changes after each titration step of $MgCl_2$ to a destacked thylakoid solution with a concentration of 10 µg Chl/mL or 30 µg Chl/mL. Under these conditions, the thylakoid concentration does not alter the response to the $Mg^{2+}$ concentration.

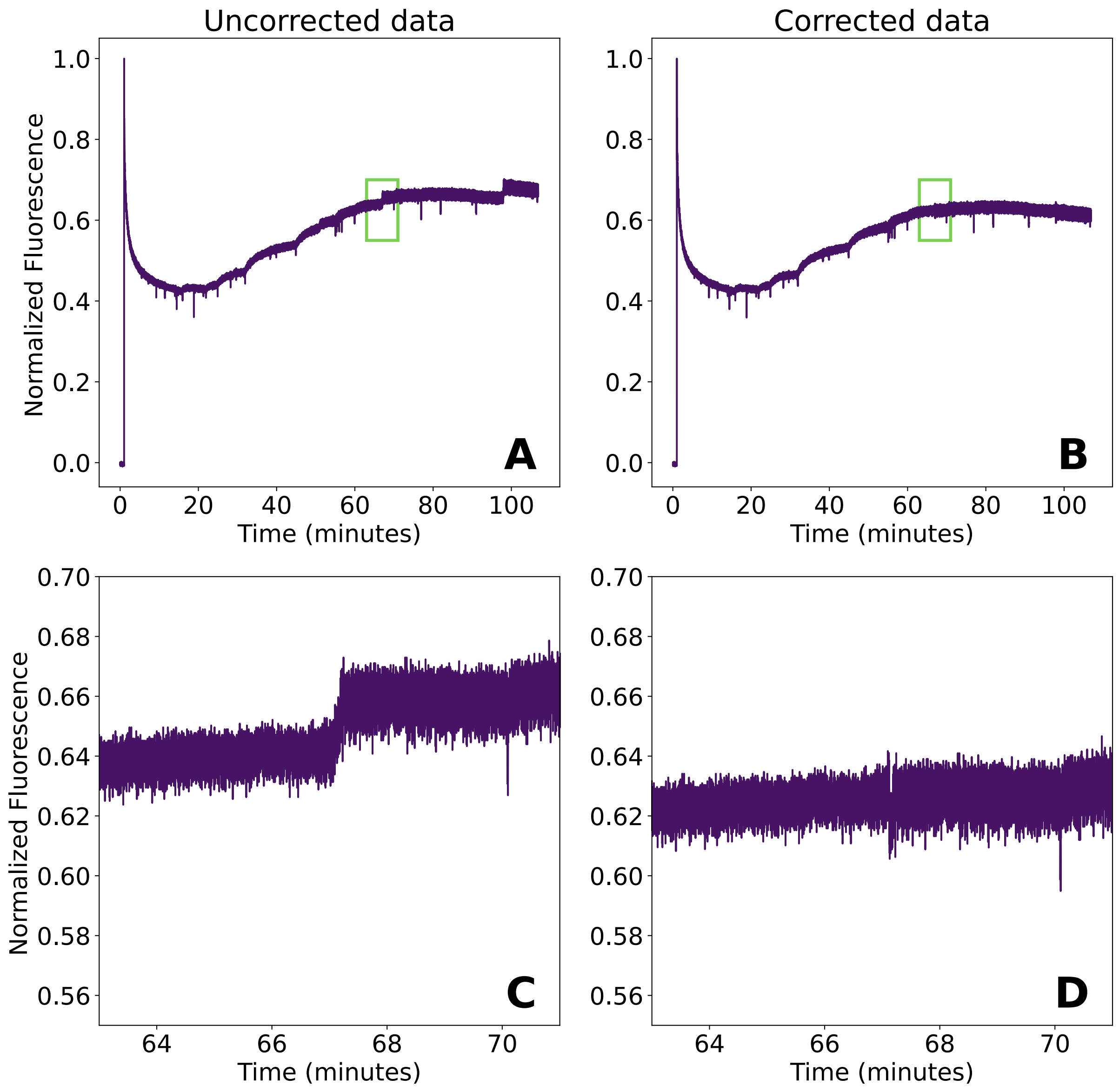


**Figure S9.** Example of fluorescence trace correction. (A) Uncorrected fluorescence trace as obtained via pulse-amplitude modulation fluorometry measurements upon thylakoid destacking and stepwise restacking. (B) Corrected fluorescence trace from (A). Signal increases due to sampling were corrected by equilibrating the average signal around the sampling timepoint. (C) Detail of the green box in (A), showing an example of an increase in fluorescence signal upon sampling. (D) Detail of the green box in (B), where the signal increase is corrected.
